# Structure-based discovery of the first non-covalent inhibitors of *Leishmania major* tryparedoxin peroxidase by high throughput docking

**DOI:** 10.1038/srep09705

**Published:** 2015-05-07

**Authors:** Margherita Brindisi, Simone Brogi, Nicola Relitti, Alessandra Vallone, Stefania Butini, Sandra Gemma, Ettore Novellino, Gianni Colotti, Gabriella Angiulli, Francesco Di Chiaro, Annarita Fiorillo, Andrea Ilari, Giuseppe Campiani

**Affiliations:** 1 European Research Centre for Drug Discovery and Development (NatSynDrugs), University of Siena, via Aldo Moro 2, 53100, Siena; 2 Dip. di Biotecnologie, Chimica e Farmacia, University of Siena, via Aldo Moro 2, 53100, Siena, Italy; 3 Dip. di Farmacia, University of Naples Federico II, Via D. Montesano 49, 80131 Naples, Italy; 4 Istituto Pasteur Fondazione Cenci-Bolognetti and Istituto di Biologia e Patologia Molecolari IBPM– CNR, c/o Dipartimento di Scienze Biochimiche, Sapienza Università di Roma Piazzale A. Moro 5, 00185 Roma (Italy); 5 Dipartimento di Scienze Biochimiche, Sapienza Università di Roma, Piazzale A. Moro 5, 00185 Roma (Italy)

## Abstract

Leishmaniasis is a neglected vector-born disease caused by a protozoan of the genus *Leishmania* and affecting more than 1.300.000 people worldwide. The couple tryparedoxin/tryparedoxin peroxidase is essential for parasite survival in the host since it neutralizes the hydrogen peroxide produced by macrophages during the infection. Herein we report a study aimed at discovering the first class of compounds able to non-covalently inhibit tryparedoxin peroxidase. We have solved the high-resolution structure of Tryparedoxin peroxidase I from *Leishmania major (Lm*TXNPx) in the reduced state and in fully folded conformation. A first series of compounds able to inhibit *Lm*TXNPx was identified by means of the high throughput docking technique. The inhibitory activity of these compounds was validated by a Horseradish peroxidase-based enzymatic assay and their affinity for *Lm*TXNPx calculated by surface plasmon resonance experiments. On the basis of these results, the analysis of the enzyme-inhibitor docked models allowed us to rationally design and synthesize a series of *N*,*N*-disubstituted 3-aminomethyl quinolones. These compounds showed an inhibitory potency against *Lm*TXNPx in the micromolar range. Among them, compound 12 represents the first non-covalent *Lm*TXNPx inhibitor reported to date and could pave the way to the discovery of a new class of drugs against leishmaniasis.

Leishmaniases are a class of infectious diseases caused by protozoan parasites of the genus *Leishmania*, transmitted through the bite of phlebotomine sandflies. According to the World Health Organization, visceral leishmaniasis kills more than 20000 people every year and over 310 million people are believed to be at risk of infection^1^. Treatment of these diseases is unsatisfactory in terms of safety and efficacy, which sharply contrasts with the therapeutic need in terms of number of people at risk, affected patients, and associated fatalities. This discrepancy is primarily due to the prevalence of these diseases in poor tropical and subtropical countries whose drug development capacity is limited. As a consequence, only a small number of drugs are available, namely antimony-containing compounds (Pentostam) and miltefosine. Both of these drugs present severe side effects and miltefosine is too expensive to be used in underdeveloped countries^2^. Therefore, there is an urgent need to develop new efficacious, less toxic and more affordable drugs.

The trypanothione-dependent hydroperoxide metabolism, characteristic of Trypanosomatids, has been recognized as a promising target for anti-leishmanial drug development since it is absent in the host and most of its components are essential to parasite survival^3^. Indeed, the defense of these parasites against the reactive oxygen species (ROS) produced by macrophages during the infection depends on the enzymes involved in the unique dithiol trypanothione (*N1,N8*-bis(glutathionyl)spermidine, T(SH)_2_) metabolism. T(SH)_2_ is synthesized by the Trypanothione Synthetase (TryS) and maintained in the reduced state by Trypanothione Reductase (TR)^4^,^5^. T(SH)_2_ participates in crucial thiol-disulfide exchange reactions and serves as electron donor in the reduction of hydrogen peroxide to water catalyzed by the tryparedoxin/tryparedoxin peroxidase couple (TXN/TXNPx). *Leishmania* TXNPx is a particularly interesting drug target. On the one hand, TXNPx has been proven to be necessary for parasite survival by gene disruption in the *Leishmania infantum* amastigote; on the other, TXNPx overexpression in *Leishmania chagasi* (*L. infantum*) confers H_2_O_2_, *t*-butyl hydroperoxide and peroxynitrite resistance^6^,^7^.

Leishmania major TXNPx (*Lm*TXNPx) is an obligate homodimer whose active site is formed by the N-proximal peroxidatic cysteine (Cp) from one subunit and a C-proximal resolving cysteine from the two-fold symmetry related subunit (Cr′)^8^,^9^. According to the generally accepted mechanism, the hydrogen peroxide reduction catalyzed by TXN/TXNPx couple consists of two distinct reactions. In the first reaction, oxidized TXNPx binds TXN, which reduces the inter-subunit disulfide bridge (Cp–Cr′) and, thereafter, is reduced by T(SH)_2_. In the second reaction, the Cp thiolate of a TXNPx monomer reduces the hydrogen peroxide to water and is oxidized to sulfenic acid (-SOH); the sulfenic Cp then reacts with the Cr′ of the two-fold symmetry related monomer forming a disulfide bridge. The transition of 2-Cys peroxiredoxin from reduced to oxidized state is associated with a conformational change involving the protein regions where Cp and Cr′ are located, namely the Cp loop and the C-terminal arm, respectively. Usually peroxiredoxins in reduced state assume the so-called fully folded (FF) conformation, where: i) Cp is part of an α-helical turn, and is oriented toward the catalytic cavity; and ii) the C-terminal tail is partially folded in an α-helix, and covers the catalytic cavity. Protein oxidation promotes a transition to a locally unfolded (LU) conformation, where: i) the α-helical turn containing Cp unwinds and rotates toward the solvent; and ii) the C-terminal tail unfolds and becomes disordered. The transition from FF to LU conformation is essential for catalysis in most peroxiredoxins, and has been related to changes in protein quaternary structure, from a decameric assembly in FF conformation to a dimeric one in LU conformation^10^. Recently Fiorillo *et* *al.*^11^ have shown by gel-filtration experiments that *Lm*TXNPx displays a decameric assembly in both the oxidized and the reduced states, and that the LU conformation is the most populated within the low resolution crystal structure of *Lm*TXNPx (PDB ID: 3TUE)^11^ obtained under reducing conditions. In this structure, the C-terminal region (residues 169–199) is not visible, even if Cp is reduced. This finding is unusual but not unprecedented since human peroxiredoxin 4 in the reduced state may adopt mixed conformations (PDB ID: 3TKQ)^12^ and in the structure of *Crithidia fasciculata* TXNPx in the reduced state (PDB ID: 1E2Y)^9^ only three out of the ten monomers forming the decameric assembly assume a correct FF conformation. Conversely, the structure of peroxiredoxin in the oxidized state has been always found to assume the LU conformation. Therefore, structural data suggest that while peroxiredoxins in the oxidized state do assume the LU conformation, peroxiredoxins in reduced state may assume both the FF and LU conformations.

In this paper, we report the X-ray crystal structure of *Lm*TXNPx in FF conformation at high resolution. Structure-based high-throughput docking to the available crystal structures of *Lm*TXNPx identified several compounds as potential enzyme inhibitors. Compounds able to inhibit the activity of TXNPx in an enzymatic assay were characterized in detail by surface plasmon resonance. Based on both the resulting inhibitory activity and examination of the docking models, a family of *N*,*N*-disubstituted 3-aminomethyl quinolone derivatives (**1–12**, Figure 1) was rationally designed, synthesized and experimentally characterized. As a result of this work, we discovered the first non-covalent inhibitors of TXNPxs reported to date. These represent suitable leads for the development of novel, effective and safer anti-leishmania drugs.

## Results and Discussion

### X-ray crystal structure study

We report the high resolution (2.34 Å) three-dimensional (3D) structure of *Lm*TXNPx in the reduced form and FF conformation (*Lm*TXNPs-FF; PDB ID: 4K1F). We previously reported a lower resolution (3.00 Å) structure of the same protein in the reduced form and LU conformation (*Lm*TXNPs-LU; PDB ID: 3TUE)^11^. The crystallization conditions used to obtain the two structures were slightly different (see materials and methods), and the unit cell of the *Lm*TXNPx crystal in FF conformation is 6% larger. The significant gain in resolution may be ascribed to the reduced flexibility of the 169–199 region, which is fully visible in the FF conformation structure here presented and absent in the previously reported LU conformation structure.

**Figure 2:**
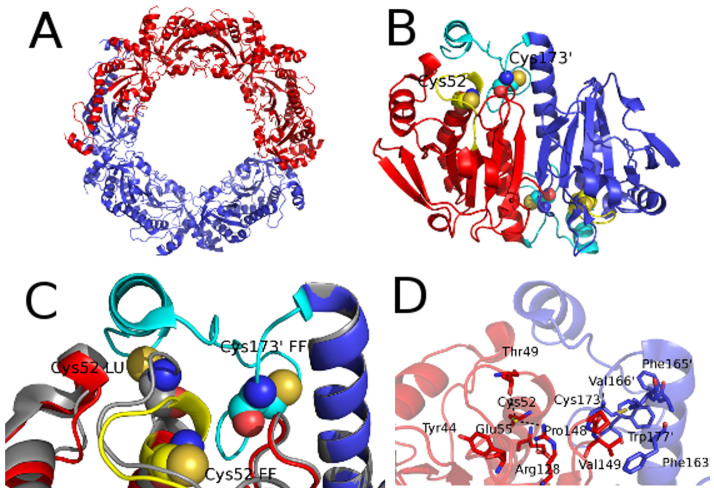
*Lm*TXNPx structure in fully folded conformation. (A). Decameric assembly of *Lm*TXNPx. Two pentamers belonging to adjacent asymmetric units are colored in red and blue respectively. (B). Functional dimer. The two fold symmetry-related subunits are indicated by different colors (red and blue). The residues of the C-terminal region (169–199) are indicated in cyan, the residues of the Cp loop in yellow. The catalytic cysteines Cp (Cys52) and Cr′ (Cys173) are depicted as spheres. (C). Superimposition between *Lm*TXNPx in FF (4K1F) and LU (3TUE) conformations: blow-up of the catalytic site. The *Lm*TXNPx-LU monomers of the dimer are depicted in orange whereas the *Lm*TXNPx-FF monomers are depicted in red and blue, respectively. The residues of the C-terminal region (169–199), visible only in the FF conformation, are indicated in cyan and the residues of the Cp loop in FF conformation in yellow. The catalytic cysteines are indicated and depicted as spheres. (D). Catalytic cysteines moiety. The catalytic cysteines, the residues surrounding and interacting with the catalytic cysteines are indicated and depicted as sticks.

The overall structure of *Lm*TXNPx-FF is very similar to that of *Lm*TXNPx-LU. Both quaternary structures are pentamers of dimers assembled to form a toroidal ring with an outer diameter of 120 Å and an internal diameter of about 60 Å (Figure 2A, B).

Optimal structure superimposition between *Lm*TXNPx-FF and *Lm*TXNPx-LU highlights that conformational changes occur just in two regions: the 43–53 loop containing Cp (Cys52) and the C-terminal tail (residues 169–199) containing Cr′ (Cys173′). In *Lm*TXNPx-FF Cp is part of the first turn of an α-helix, located in a narrow solvent-accessible pocket that constitutes the active site. Conversely, in *Lm*TXNPx-LU the helix partially unwinds and Cp becomes completely exposed to the solvent and available to be attacked by Cr′ to form a disulfide bridge (Figure 2B, C). As shown in Figure 2 (panels C and D), Cp is hydrogen bonded to the highly conserved Thr49 and Arg128 residues, both of which are essential for catalysis^9^,^10^. The C-terminal tail (residues 169–199), which is missing in *Lm*TXNPx-LU, is completely visible in *Lm*TXNPx-FF, thus revealing the complete active site structure of the protein (Figure 2C). Cr′ is part of a loop and its side chain is embedded in a hydrophobic cavity formed by the 170–187 residues. The entrance to this active site cavity is covered by the short C-terminal α-helix (residues 187–195), which protects Cr′ from over-oxidation. Interestingly, different crystallization conditions selected for either *Lm*TXNPx-FF or *Lm*TXNPx-LU, both of which were demonstrated to be present under reducing conditions. Since the oxidized *Lm*TXNPx state is generally associated with the LU conformation, structure comparison between the here reported *Lm*TXNPx-FF and the previously solved *Lm*TXNPx-LU structures provided us with the opportunity to infer the mechanism underlying the transition from FF to LU conformation that occurs upon protein oxidation. Reduced *Lm*TXNPx-FF is ready to exert its catalytic function, i.e., reduction of hydrogen peroxide to water, involving oxidation of Cp to sulphenic acid. Upon Cp oxidation, *Lm*TXNPx undergoes a local conformational change (Figure 2C), consisting in the unwinding of the α-helical turn comprising Cp and disordering of the last 20 residues of the protein comprising Cr′, and resulting in exposure to the solvent of both catalytic cysteines.

Additionally, structural features from both the complete *Lm*TXNPx-FF structure and the *Lm*TXNPx-LU counterpart were exploited for subsequent molecular dynamics and docking studies, and to design specific molecules able to inhibit the enzyme catalytic activity.

### High-throughput docking

In order to select potential inhibitors of *Lm*TXNPx, a high-throughput docking (HTD) procedure was employed^13^. Both *Lm*TXNPx-LU and *Lm*TXNPx-FF crystal structures were exploited by the HTD procedure (GOLD software, Genetic Optimization for Ligand Docking)^14^,^15^. First, both structures were analyzed to find suitable binding sites for HTD screening. The SiteMap program (SiteMap, version 2.5, Schrödinger, LLC, New York, NY, 2011) identified a potential large binding site in the *Lm*TXNPx-LU structure (Figure S1). However, access to this binding site might in principle be restricted by the C-terminal protein region, which is not visible in the X-ray structure due to a disordered conformation. In *Lm*TXNPx-FF access to the above-mentioned binding site is hampered by the C-terminal tail, such that a very small volume is solvent accessible (88.39 Å^3^, Figure S2A). Since no binding sites suitable for in silico screening were observed in the crystal structures, the complete *Lm*TXNPx-FF structure was used for Molecular Dynamics (MD) simulations using Desmond software (Desmond Molecular Dynamics System, version 3.0, D. E. Shaw Research, New York, NY, 2011. Maestro-Desmond Interoperability Tools, version 3.0, Schrödinger, New York, NY, 2011.). MD results also suggested a high flexibility of the C-terminal tail of the protein (Figure S3) and predicted that a conformational shift to a larger binding site (volume = 233.37 Å^3^) occurred in the protein (Figure S2B). The results of a study performed with the CABS-flex server (http://biocomp.chem.uw.edu.pl/CABSflex/index.php), which implements one of the best algorithms specialized in residue fluctuation analysis, were also in agreement with the flexibility of the C-terminal tail (data not shown). From the above 20 ns MD simulation we selected the first frame, whose binding site volume was comparable to that present in the *Lm*TXNPx-LU structure. This “open” binding site was used for docking studies (see experimental section for further details). The HTD campaign was performed using two chemical libraries, one of which proprietary (containing around 2,300 small molecules) and the other commercial (Asinex Ltd., 5 Gabrichevskogo, St. Building 8, Moscow 125367, Russia) containing around 250,000 small molecules. The scoring function GoldScore was employed to rank the compounds best fitting the predicted binding site. Based on our protocol (see experimental section for further details) twenty potential *Lm*TXNPx ligands were selected and submitted to biochemical evaluation (Table S1). The selection was performed applying a GoldScore cutoff of 65 coupled to cluster analysis and visual inspection.

### Evaluation of inhibitory activity by enzymatic assay

To determine the ability of the selected compounds to inhibit the catalytic activity of *Lm*TXNPx, we used the horseradish peroxidase (HRP)-H_2_O_2_ competition assay developed by Augusto and co-workers^16^. HRP is well known to react with H_2_O_2_ in a two-electron oxidation process that leads to the formation of HRP compound I (HRP-I), a stable HRP oxidized intermediate that can be followed at 398 nm. Reduced *Lm*TXNPx competes with HRP for the H_2_O_2_ substrate, leading to a decrease in HRP-I yield that is proportional to *Lm*TXNPx concentration. The addition of *Lm*TXNPx inhibitors leads to a decrease in free *Lm*TXNPx concentration and consequent increase in the HRP-I yield.

Six out of twenty tested compounds exhibited significant inhibitory activity against *Lm*TXNPx at 100 μM (Table S1).

### Design and synthesis of optimized compounds

**Figure 3:**
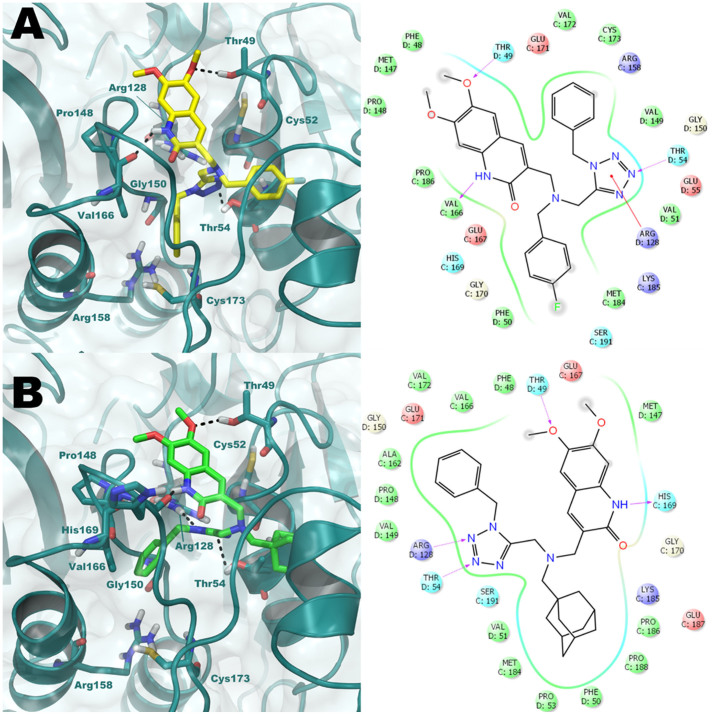
Putative binding mode of 1 (A, yellow sticks) and 12 (B, green sticks) obtained by GOLD software (GoldScore values 70.49 and 78.91 for 1 and 12, respectively) into predicted active site of the *Lm*TXNPx-FF enzyme (deep teal cartoon). The key residues of the binding site of the enzyme are represented by sticks. H-bonds are reported by grey dotted lines. The picture was generated by means of PyMOL. Ligand-interaction diagrams are generated by Maestro.

The predicted orientation of representative compounds **1** and NF1442 within the *Lm*TXNPx obtained by the MD and HTD simulations described above are reported in Figures 3A in the Main Text and S4 in SI, respectively. Notably, in these models, both compounds were able to interact with residues that have been demonstrated to be crucial for catalysis. In particular, compound **1** (Figure 3A and Table 1) forms H-bonds with Thr49, Thr54 and Val166 and a cation-π interaction with Arg128. For comparison, we docked inhibitor **1** in *Lm*TXNPx-LU binding site (Figure S5 in the SI). In both binding poses the inhibitor occupies the same binding pocket, interacting with the same key residues. However, the *p*-fluorophenyl ring of **1** in the *Lm*TXNPx-LU structure is solvent exposed (Figure S5), while it interacts with specific residues of the C-terminal tail in the MD frame of the *Lm*TXNPx-FF structure (Figure 3A). SAR studies (see below) are consistent with the proposed binding mode of compound **1**. To better investigate the interaction of inhibitor **1** with the highly flexible C-terminal tail, we have submitted the complex **1**/*Lm*TXNPx-FF to further 20 ns of MD simulation, starting from the docking pose reported in Figure 3A and following a protocol previously applied for different targets^17^,^18^,^19^. In the snapshot of the trajectory reported in Figure S6 in the SI, compound **1** directly interacts with Cys173 of the loop at the C-terminal region forming a H-bond interaction from the half to the end of the simulation. Moreover, the compound binds deeper in the binding site, losing the H-bond interactions observed in the previous docking in favor of H-bonding to Gly150 and Arg128. A cation-π stacking with Arg158 is also found. These events block the enzyme functioning since the binding of **1** physically hampers the correct C-terminal tail accommodation. A preliminary ligand optimization study was performed and suitable sites for molecular decoration of the hit (compounds **2–12**, Table 1) were identified.

**Figure 4:**
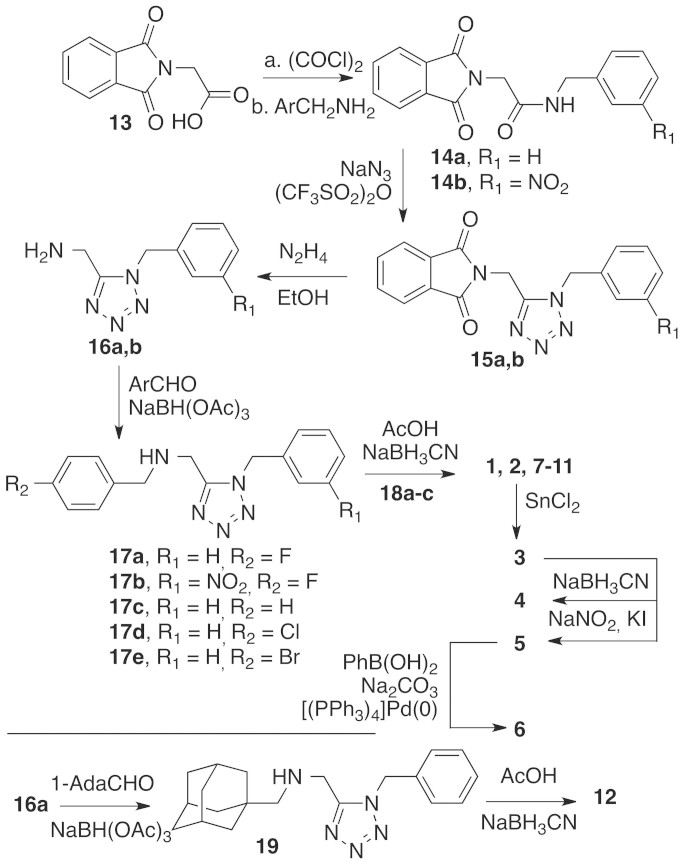
Synthesis of Inhibitors 1–12.

The synthesis of inhibitors **1**–**12** is reported in Figure 4. The carboxylic acid **13** was activated as the corresponding acyl chloride and then reacted with the suitable benzylamines. Amides **14a,b** thus obtained were submitted to a cyclization protocol in the presence of sodium azide and trifluoromethanesulfonic anhydride to afford tetrazoles **15a,b**. Hydrazinolysis of the phthalimide moiety afforded primary amines **16a,b** that where subsequently submitted to a reductive amination protocol employing the appropriately substituted benzaldehydes in the presence of sodium triacetoxyborohydride. Secondary amines **17a–e** were then used as substrates of a second reductive amination protocol using quinoline carboxaldehydes **18a–c** (synthesized as described in Scheme S1), affording the final compounds **1**, **2** and **7–11**. The nitro group of compound **2** was reduced to the corresponding aniline by treatment with tin (II) chloride in ethanol to afford inhibitor **3**. The amino group of this latter compound was methylated by using an excess of formaldehyde in the presence of sodium cyanoborohydride as the reducing agent to afford inhibitor **4**. Starting from **3**, inhibitor **5** was synthesized using the Sandmeyer reaction. Starting from the iodide derivative **5**, a Suzuki coupling with phenylboronic acid catalyzed by tetrakis(triphenylphosphine)palladium(0) provided the biphenyl derivative **6**. Finally, inhibitor **12** was synthesized by reacting intermediate **15a** with 1-adamantylcarboxaldehyde to obtain **12**, which was then submitted to the second reductive amination reaction as previously described.

### Quantitative evaluation of inhibitory activity by surface plasmon resonance and structure-activity relationships

The method of HRP-based competition assay^16^,^20^ was used only to establish if the compounds were able to inhibit *Lm*TXNPx. It was not possible to measure directly the *K*_i_ with this method since the experiments were not carried out in a steady-state condition and on the other hand the experimental data are difficult to fit due to the presence of different error sources as detailed in the Methods session (see below). To determine the *K*_i_ of the enzyme we set up a high-throughput method which combines the HRP-H_2_O_2_ competition assay, according to Augusto and co-workers^16^, with the SPR experiments (*K*_D_ values, Table 1 and Figure S7 in the SI). All synthesized compounds showed good to reasonable degrees of enzyme inhibition with the exception of **9** and **10**. Indeed, it was not possible to evaluate the ability of these latter compounds to inhibit *Lm*TXNPx since they interfere with the inhibition assay by interacting with HRP. The affinity of the compounds for the hypothesized binding site was improved for all compounds with the exception of **3**. The experimental data are consistent with the calculated docking score and free binding energy (ΔG_bind_) (Table S2, Figure S8 and SAR analysis discussion in the SI). We firstly placed specific substituents at the *m*-position of the unsubstituted aromatic ring of **1**. Accordingly, docking studies predicted that substituents at this position could establish favorable interactions with the protein. However, decoration of the aromatic ring with nitro (**2**), amino (**3**), dimethylamino (**4**), and iodine (**5**) did not result in a marked increase of binding affinity since the extra-interactions of these substituents slightly shifted the correct orientation of the whole scaffold. This was particularly evident for compound **3** in which the aniline forms a H-bond with the backbone of Glu171 but the key interaction with Thr49 is lost as well as the correct orientation of the *p*-fluorophenyl ring in the hydrophobic cavity. On the other hand, introduction of an aromatic ring at this position led to one of the most potent hit of the series (**6**, *K*_D_ = 52 μM). Accordingly, the extra-phenyl ring occupies a hydrophobic sub-pocket and forms a strong interaction with Arg158 by a double π-π stacking. Secondly, we modified the substitution pattern of the quinolone ring by removing the 7-OMe group, which, according to our computational predictions, is not involved in H-bond interactions with the enzyme. Gratifyingly, the resulting 6-OMe derivative **7** showed an improved *K*_D_ with respect to our early hit **1** as predicted by the calculated (ΔG_bind_). The improved activity can be explained by the formation of stronger interactions with Arg128 and Thr54. Notably, removing both OMe groups from the quinolone ring (**8**) resulted in a decrease in enzyme affinity due to an overall alteration of the binding mode. In a further round of SARs, we assessed the role of the fluorine substituent on the second aromatic ring of **1** by replacing it with H (**9**), Cl (**10**) and Br (**12**); all these modifications resulted in an increase of affinity. From our docking studies, removal of the F substituent allows the formation of a stacking interaction with Phe50. No relevant differences were found with the replacement of F with Cl and Br since both compounds showed a similar binding mode. However, inhibitor **10** forms better interactions with His169 and Thr54 than **11**. The best compound of the series was identified when the *p*-fluorophenyl ring was replaced by a bulky aliphatic adamantyl system (inhibitor **12**). The docking output of **12** is reported in Figure 3B. Compound **12** shows a binding mode comparable to that found for the hit compound **1**, and it is also able to form a H-bond with the backbone of His169, preserving the other H-bond interactions previously observed in the docking of **1**. Replacement of the fluorophenyl ring with an adamantyl moiety resulted in the formation of optimized hydrophobic contacts with Val51, Val128, Ile78 Pro186, Pro188, Pro53 and Phe50. In silico calculation performed by QikProp (Table S3) showed that this class of compounds presents interesting predicted drug-like properties such as *c*logP, *c*logS and absorption.

## Conclusions

To the best of our knowledge, this paper describes the first non-covalent inhibitors of tryparedoxin peroxidase I of Leishmania parasite. These were developed by a comprehensive approach encompassing X-ray structure determination and a combination of HTD and preliminary hit optimization. Compound **12** was identified as the most effective in both the HRP-H_2_O_2_ competition assay and SPR experiments.

Upon optimization, these non-covalent inhibitors could pave the way to the discovery of new potential drugs against leishmaniasis. Moreover, the combination of the HRP-H_2_O_2_ assay with SPR measurements appears to be an effective strategy to obtain complementary qualitative information about the compound ability to inhibit enzyme activity and quantitative thermodynamic parameters describing enzyme binding. Studies aimed at obtaining more potent inhibitors that might be tested against leishmania in vitro are currently ongoing.

## Methods

### Chemistry

#### General methods

All starting chemicals and solvents were purchased from commercial vendors and used without additional purification. Silica gel 60 F254 (0.040–0.063 mm) with UV detection was used for checking the reaction progress. Column chromatography was performed on silica gel 60 (0.040–0.063 mm). ^1^H NMR and ^13^C NMR spectra were recorded on a Varian 300 MHz or Bruker 400 MHz spectrometer. The residual signal of the deuterated solvent was used as internal standard. Splitting patterns are expressed as singlet (s), doublet (d), triplet (t), quartet (q), and broad (br); the value of chemical shifts (δ) are given in ppm and coupling constants (*J*) in hertz (Hz). Mass spectra were recorded utilizing electron spray ionization (ESI). Yields refer to purified products and are not optimized. All reactions were run in an inert atmosphere using oven-dried glassware and anhydrous solvents. Combustion analysis (CHN) was used to confirm the purity (>95%).

#### N-Benzyl-2-(1,3-dioxoisoindolin-2-yl)acetamide (**14a**)

To a solution of **13** (1.0 g, 4.9 mmol) in dry toluene (25 mL), oxalyl chloride (850 μL, 9.8 mmol) was added dropwise. The reaction was heated to 90 °C for 12 h under Ar atmosphere. Volatiles were removed in vacuo, and crude chloride was used in the next step without further purification. ^1^H NMR (300 MHz, CDCl_3_) δ 7.96–7.87 (m, 2H), 7.83–7.74 (m, 2H), 4.82 (s, 2H). The resulting chloride (1.1 g, 4.9 mmol) was dissolved in dry DCM (25 mL), and benzylamine (1.1 mL, 9.8 mmol) was added. The reaction was stirred at 25°C for 5 min. The solid obtained was collected giving **14a** as a white solid (1.2 g, 84%). ^1^H NMR (300 MHz, CDCl_3_) δ 8.10–7.84 (m, 2H), 7.84–7.58 (m, 2H), 7.52–7.07 (m, 5H), 5.99 (br s, 1H), 4.49 (d, *J* = 5.5 Hz, 2H), 4.39 (s, 2H). MS (ESI) *m*/*z* 293 [M-H]^−^.

#### 2-(1,3-Dioxoisoindolin-2-yl)-N-(3-nitrobenzyl)acetamide (**14b**)

Starting from **13** (1.0 g, 4.9 mmol) the corresponding chloride was obtained following the procedure described for **14a**. ^1^H NMR (300 MHz, CDCl_3_) δ 7.96–7.87 (m, 2H), 7.83–7.74 (m, 2H), 4.82 (s, 2H). The obtained chloride (950 mg, 4.3 mmol) was added to a solution of 3-nitrobenzylamine hydrochloride (1.2 g, 6.4 mmol) and TEA (1.8 mL, 12.8 mmol) in dry DCM (50 mL). The reaction was stirred at 25 °C for 3 h under Ar atmosphere. The solid formed was collected, giving **14b** as a brown solid (1.3 g, 90%). ^1^H NMR (400 MHz, DMSO-*d*_6_) δ 8.86 (s, 1H), 8.26–7.99 (m, 1H), 7.99–7.75 (m, 4H), 7.75–7.42 (m, 2H), 4.39 (d, *J* = 5.7 Hz, 2H), 4.25 (s, 2H). MS (ESI) *m*/*z* 338 [M-H]^−^.

#### 2-((1-Benzyl-1H-tetrazol-5-yl)methyl)isoindoline-1,3-dione (**15a**)

To a stirred solution of **14a** (500 mg, 1.7 mmol) in CH_3_CN (60 mL), NaN_3 _(326 mg, 5.0 mmol) and trifluoromethanesulfonic anhydride (1.7 mL, 10.2 mmol) were added at 0 °C. The reaction was allowed to reach 25 °C and stirred for 12 h under Ar atmosphere. A saturated solution of NaHCO_3_ was added, CH_3_CN was evaporated in vacuo and the residue was extracted with EtOAc (3 × 20 mL). The combined organic extracts were dried over Na_2_SO_4_, filtered, and evaporated. The crude product was purified by flash chromatography on silica gel (2% MeOH in CHCl_3_) to give **15a** as a pale yellow oil (260 mg, 48%). ^1^H NMR (300 MHz, CDCl_3_) δ 7.89–7.60 (m, 4H), 7.36–7.01 (m, 5H), 5.73 (s, 2H), 4.97 (s, 2H). MS (ESI) *m*/*z* 320[M + H]^+^.

#### 2-((1-(3-Nitrobenzyl)-1H-tetrazol-5-yl)methyl)isoindoline-1,3-dione (**15b**)

Starting from **14b** (870 mg, 2.6 mmol), the title compound was prepared following the procedure reported for **15a**. The crude material was purified by flash chromatography on silica gel (2% MeOH in CHCl_3_) to give **15b** as a yellow solid (500 mg, 53%). ^1^H NMR (300 MHz, CDCl_3_) δ 8.20–7.91 (m, 2H), 7.90–7.61 (m, 4H), 7.61–7.33 (m, 2H), 5.84 (s, 2H), 5.10 (s, 2H). MS (ESI) *m*/*z* 387 [M + Na]^+^.

#### 1-Benzyl-1H-tetrazol-5-y*lm*ethanamine (**16a**)

To a stirred solution of **15a** (260 mg, 0.8 mmol) in EtOH (30 mL), hydrazine monohydrate (160 μL, 3.4 mmol) was added. The reaction was refluxed under Ar atmosphere for 1.5 h. A 2 N solution of NaOH was added, EtOH was evaporated in vacuo and the residue was extracted with DCM (3 × 10 mL). The combined organic extracts were dried over Na_2_SO_4_, filtered, and evaporated under reduced pressure. The crude product was purified by flash chromatography on silica gel (5% MeOH in DCM) to give **16a** as a colorless oil (110 mg, 71%). ^1^H NMR (400 MHz, CDCl_3_) δ 6.65–6.27 (m, 3H), 6.27–6.18 (m, 2H), 4.66 (s, 2H), 3.02 (s, 2H), 0.56 (br s, 2H). MS (ESI) *m*/*z* 212 [M + Na]^+^.

#### (1-(3-Nitrobenzyl)-1H-tetrazol-5-yl)methanamine (**16b**)

Starting from **15b** (150 mg, 0.4 mmol) the title compound was prepared following the procedure reported for compound **16a**. The crude product was purified by flash chromatography on silica gel (5% MeOH in DCM) to give **16b** as a yellow oil (91 mg, 95%). ^1^H NMR (300 MHz, CDCl_3_) δ 8.21–7.83 (m, 2H), 7.57 (d, J = 7.7 Hz, 1H), 7.44 (t, J = 7.9 Hz, 1H), 5.73 (s, 2H), 4.06 (s, 2H), 1.68 (br s, 2H). MS (ESI) m/z 235[M + H]^+^, 257 [M + Na]^+^.

#### (Benzyltetrazolyl)-N-(4-fluorobenzyl)methanamine (**17a**)

To a solution of **16a** (46.0 mg, 0.2 mmol) in dry DCM (6.0 mL), 4-fluoro-benzaldehyde (20 μL, 0.19 mmol) was added, then Na(OAc)_3_BH (58 mg, 0.27 mmol) was added at 0°C and the mixture kept at 25 °C for 12 h. After this time NaCNBH_3_ (17 mg, 0.27 mmol) was added and the solution was maintained at the same temperature for further 30 min. A saturated solution of NaHCO_3_ was added, and the mixture was extracted with DCM (3 × 2 mL), dried over Na_2_SO_4_, filtered, and evaporated in vacuo. The crude material was purified by flash chromatography on silica gel (2% MeOH in CHCl_3_) to give **17a** as colorless oil (51 mg, 73%). ^1^H NMR (CDCl_3_): δ 7.33–7.30 (m, 3H), 7.21–7.14 (m, 4H), 7.02–6.96 (m, 2H), 5.72 (s, 2H), 3.70 (s, 2H), 3.61 (s, 2H), 1.95 (br s, 1H). MS (ESI) *m*/*z* 299 [M + H]^+^; 321 [M + Na]^+^.

#### N-(4-Fluorobenzyl)-1-(1-(3-nitrobenzyl)-1H-tetrazol-5-yl)methanamine (**17b**)

Starting from **16b** (380 mg, 1.6 mmol) the title compound was prepared following the same procedure of **17a**. The crude product was purified by flash chromatography on silica gel (20% PetEt in EtOAc) to give **17b** as a yellow solid (450 mg, 82%). ^1^H NMR (300 MHz, CDCl_3_) δ 8.37–7.96 (m, 2H), 7.54 (d, *J* = 4.8 Hz, 2H), 7.32–7.12 (m, 2H), 7.01 (t, *J* = 8.6 Hz, 2H), 5.72 (s, 2H), 4.03 (s, 2H), 3.75 (s, 2H). MS (ESI) *m*/*z* 343 [M + H]^+^; 365 [M + Na].

#### (Benzyltetrazolyl)-N-(benzyl)methanamine (**17c**)

Starting from **16a** (27.0 mg, 0.1 mmol) and benzaldehyde (13.4 μL, 0.1 mmol) the title compound was prepared following the same procedure of **17a**. The crude material was purified by flash chromatography on silica gel (2% MeOH in CHCl_3_) to give **17c** as colorless oil (25 mg, 69%). ^1^H NMR (CDCl_3_): δ 7.39–7.22 (m, 8H), 7.18–7.09 (m, 2H), 5.62 (s, 2H), 3.93 (s, 2H), 3.73 (s, 2H), 1.92 (br s, 1H). MS (ESI) *m/z* 281 [M + H]^+^; 302 [M + Na]^+^.

#### (Benzyltetrazolyl)-N-(4-clorobenzyl)methanamine (**17d**)

Starting from **16a** (35.0 mg, 0.2 mmol) and 4-clorobenzaldehyde (24.0 mg, 0.2 mmol) the title compound was prepared following the same procedure of **17a**. The crude product was purified by flash chromatography on silica gel (2% MeOH in CHCl_3_) to give **17d** as a colorless oil (40 mg, 75%). ^1^H NMR (CDCl_3_): δ 7.31–7.25 (m, 5H), 7.18–7.12 (m, 4H), 5.60 (s, 2H), 3.90 (s, 2H), 3.69 (s, 2H), 1.87 (br s, 1H). MS (ESI) *m/z* 314 [M + H]^+^; 356 [M + Na]^+^.

#### (Benzyltetrazolyl)-N-(4-bromobenzyl)methanamine (**17e**)

Starting from **16a** (35.0 mg, 0.2 mmol) and 4-bromobenzaldehyde (31.4 mg, 0.2 mmol) the title compound was prepared following the same procedure of **17a**. The crude material was purified by flash chromatography on silica gel (2% MeOH in CHCl_3_) to give **17e** as a colorless oil (35 mg, 58%). ^1^H NMR (CDCl_3_): δ 7.45–7.32 (m, 4H), 7.13–7.10 (m, 5H), 5.60 (s, 2H), 3.90 (s, 2H), 3.69 (s, 2H), 1.90 (br s, 1H). MS (ESI) *m/z* 358 [M + H]^+^; 380 [M + Na]^+^.

#### 3-((((1-Benzyl-1H-tetrazol-5-yl)methyl)(4-fluoro benzyl)amino)methyl)-6,7-dimethoxyquinolin-2(1H)-one (**1**)

Compound **17a** (51.0 mg, 0.2 mmol) was suspended in a mixture of MeOH/AcOH 1% (5 mL). **18a** (40.0 mg, 0.2 mmol) and NaCNBH_3 _(21.0 mg, 0.4 mmol) were added and the reaction was heated to 70 °C for 12 h. A saturated solution of NaHCO_3_ was added, MeOH was evaporated under reduced pressure, and the aqueous phase was extracted with DCM (3 × 2 mL). Combined organic extracts were dried over Na_2_SO_4_, filtered, and concentrated in vacuo. The crude material was purified by flash chromatography on silica gel (33% DCM in CH_3_CN) to give **1** as a colorless oil (40 mg, 50%). ^1^H NMR (CDCl_3_): δ 12.72 (br s, 1H), 7.66 (s, 1H), 7.30–7.26 (m, 2H), 7.15–7.07 (m, 3H), 7.00–6.95 (m, 2H), 6.88 (s, 1H), 6.77–6.80 (m, 3H), 5.44 (s, 2H), 3.91 (s, 5H), 3.89 (s, 3H), 3.76 (s, 2H), 3.73 (s, 2H). MS (ESI) *m/z* 537 [M + Na]^+^; analysis (calcd., found for C_28_H_27_FN_6_O_3_): C (65.36, 65.29), H (5.29, 5.69), N (16.33, 16.08).

#### 3-(((4-Fluorobenzyl)((1-(3-nitrobenzyl)-1H-tetrazol-5-yl)methyl)amino)methyl)-6,7-dimethoxyquinolin-2(1H)-one (**2**)

Starting from **17b** (450 mg, 1.3 mmol) the title compound was prepared following the procedure of **1**. The crude product was purified by flash chromatography on silica gel (EtOAc) to give **2** as a yellow solid (370 mg, 51%). ^1^H NMR (300 MHz, DMSO-*d*_6_) δ 11.52 (br s, 1H), 8.07 (d, *J* = 6.7 Hz, 1H), 7.96 (s, 1H), 7.64 (s, 1H), 7.56–7.37 (m, 2H), 7.37–7.14 (m, 2H), 7.14–6.85 (m, 3H), 6.76 (s, 1H), 5.75 (s, 2H), 4.01 (s, 2H), 3.77 (s, 3H), 3.75 (s, 3H), 3.65 (s, 2H), 3.52 (s, 2H). ^13^C NMR (75 MHz, DMSO-*d*_6_) δ 163.6, 162.3, 160.4, 153.8, 152.3, 148.3, 145.4, 138.7, 137.2, 134.7, 134.5, 134.3, 131.7, 130.9, 126.6, 123.6, 123.0, 115.7, 115.4, 112.7, 109.3, 98.0, 57.4, 56.4, 56.2, 53.1, 49.6, 46.3. MS (ESI) *m*/*z* 582 [M + Na]^+^; analysis (calcd., found for C_28_H_26_FN_7_O_5_): C (60.10, 59.93), H (4.68, 4.35), N (17.52, 17.72).

#### 3-((((1-(3-Aminobenzyl)-1H-tetrazol-5-yl)methyl)(4-fluorobenzyl)amino)methyl)-6,7-dimethoxyquinolin-2(1H)-one (**3**)

To a stirred solution of **2** (40 mg, 0.1 mmol) in EtOH (5 mL), SnCl_2_ (81 mg, 0.4 mmol) was added. Reaction was stirred at reflux temperature for 3 h. Solution was poured on ice, and a saturated aqueous solution of NaHCO_3_ was added. The resulting suspension was filtered through celite, MeOH was evaporated in vacuo and aqueous phase was extracted with DCM (3 × 10 mL). Combined organic extracts were dried over Na_2_SO_4_, filtered, and concentrated under reduced pressure. The crude material was purified by flash chromatography on silica gel (3.3% MeOH in DCM) to give **3** as a yellow oil (35 mg, 90%). ^1^H NMR (300 MHz, CDCl_3_) δ 12.40 (br s, 1H), 7.65 (s, 1H), 7.36–7.26 (m, 2H), 6.99 (t, *J* = 8.6 Hz, 2H), 6.93–6.76 (m, 3H), 6.43 (dd, *J* = 8.0, 1.5 Hz, 1H), 6.14 (d, *J* = 7.6 Hz, 1H), 6.12–6.06 (m, 1H), 5.34 (s, 2H), 4.07–3.80 (m, 8H), 3.75 (s, 2H), 3.73 (s, 2H), 3.60 (br s, 2H). ^13^C NMR (75 MHz, CDCl_3_) δ 164.2, 164.1, 160.8, 152.9, 152.6, 147.1, 146.3, 140.2, 134.9, 134.2, 133.8, 133.7, 131.2, 131.1, 129.9, 125.4, 117.2, 115.8, 115.5, 115.3, 113.7, 113.3, 108.1, 98.02, 58.4, 56.5, 56.5, 53.0, 50.8, 45.6. MS (ESI) *m*/*z* 552 [M + Na]^+^; analysis (calcd., found for C_28_H_28_FN_7_O_3_): C (63.50, 63.66), H (5.33, 5.72), N (18.51, 18.15).

#### 3-((((1-(3-(Dimethylamino)benzyl)-1H-tetrazol-5-yl)methyl)(4-fluorobenzyl)amino)methyl)-6,7-dimethoxyquinolin-2(1H)-one (**4**)

To a solution of CH_3_CN/AcOH 1% (1.5 mL), **3** (20 mg, 0.04 mmol) and formaldehyde 37% (28 μL, 0.4 mmol) were added. The solution was stirred for 5 min, then NaCNBH_3_ (7 mg, 0.1 mmol) was added, and reaction was stirred for 1.5 h. A saturated solution of NaHCO_3_ was added, CH_3_CN was evaporated, and aqueous phase was extracted with EtOAc (3 × 5 mL). Combined organic extracts were dried over Na_2_SO_4_, filtered, and concentrated in vacuo. The crude material was purified by flash chromatography on silica gel (EtOAc) to give **4** (17 mg, 80%) as a white solid. ^1^H NMR (300 MHz, CDCl_3_) δ 12.38 (br s, 1H), 7.68 (s, 1H), 7.36–7.26 (m, 2H), 7.06–6.83 (m, 4H), 6.77 (s, 1H), 6.50 (dd, *J* = 8.3, 2.4 Hz, 1H), 6.26 (s, 1H), 6.07 (d, *J* = 7.5 Hz, 1H), 5.40 (s, 2H), 4.00–3.90 (m, 5H), 3.89 (s, 3H), 3.77 (s, 2H), 3.75 (s, 2H), 2.77 (s, 6H). MS (ESI) *m*/*z* 578 [M + H]^+^; analysis (calcd., found for C_30_H_32_FN_7_O_3_): C (64.62, 64.25), H (5.78, 5.78), N (17.58, 17.82).

#### 3-(((4-Fluorobenzyl)((1-(3-iodobenzyl)-1H-tetrazol-5-yl)methyl)amino)methyl)-6,7-dimethoxyquinolin-2(1H)-one (**5**)

To a suspension of water (500 μL), H_2_SO_4_ (12 μL, 0.4 mmol) and **3** (20 mg, 0.04 mmol) cooled at 0°C, a chilled solution of NaNO_2_ (5 mg, 0.1 mmol) in water was added. Reaction was stirred at 0 °C for 10 min, and then a chilled solution of KI (14 mg, 0.1 mmol) in water was added. Reaction was stirred for other 10 min, a solution of NaOH 1 N was added, and aqueous phase was extracted with EtOAc (3 × 2 mL). Combined organic extracts were washed with a saturated solution of Na_2_S_2_O_3_, dried over Na_2_SO_4_, filtered, and concentrated in vacuo. The crude product was purified by flash chromatography on silica gel (2% MeOH in DCM) to give **5** (23 mg, 95%) as a yellow oil. ^1^H NMR (300 MHz, CDCl_3_) δ 11.80 (br s, 1H), 7.60 (s, 1H), 7.51 (d, *J* = 7.4 Hz, 1H), 7.33–7.20 (m, 3H), 7.00 (t, *J* = 8.5 Hz, 2H), 6.93–6.73 (m, 4H), 5.43 (s, 2H), 3.93 (s, 8H), 3.74 (s, 2H), 3.69 (s, 2H). ^13^C NMR (75 MHz, CDCl_3_) δ 163.0, 162.4, 159.7, 151.9, 150.9, 145.1, 139.6, 136.6, 135.2, 134.7, 133.0, 131.4, 130.2, 130.1, 129.5, 127.9, 125.6, 114.8, 114.5, 111.9, 106.9, 96.7, 93.5, 57.1, 55.4, 55.3, 52.0, 48.8, 44.2. MS (ESI) *m*/*z* 663 [M + Na]^+^; analysis (calcd., found for C_28_H_26_FIN_6_O_3_): C (52.51, 52.22), H (4.09, 3.91), N (13.12, 13.20).

#### 3-((((1-([1,1′-Biphenyl]-3-ylmethyl)-1H-tetrazol-5-yl)methyl)(4-fluorobenzyl)amino)methyl)-6,7-dimethoxyquinolin-2(1H)-one (**6**)

To a solution of **5** (20 mg, 0.03 mmol), in toluene (2.2 mL), Pd(PPh_3_)_4_ (2 mg, 0.001 mmol), a solution of Na_2_CO_3_ (24 mg, 0.2 mmol) in water (0.7 mL), and a solution of phenylboronic acid (4 mg, 0.03 mmol) in EtOH (1 mL) were added in this order. Reaction was refluxed for 6 h, then water was added at 25 °C, solvent evaporated under reduced pressure, and aqueous phase extracted with DCM (3 × 2 mL). Combined organic extracts were dried over Na_2_SO_4_, filtered, and concentrated under reduced pressure. The crude product was purified by flash chromatography on silica gel (3.3% MeOH in DCM) to give **6** (11 mg, 60%) as a white solid. ^1^H NMR (300 MHz, CDCl_3_) δ 11.62 (br s, 1H), 7.62 (s, 2H), 7.50–7.23 (m, 7H), 7.23–7.06 (m, 2H), 6.94 (t, *J* = 7.9 Hz, 2H), 6.89–6.75 (m, 2H), 6.66 (s, 1H), 5.55 (s, 2H), 3.96 (s, 2H), 3.88 (s, 6H), 3.76 (s, 2H), 3.72 (s, 2H). MS (ESI) *m*/*z* 613 [M + Na]^+^; analysis (calcd., found for C_34_H_31_FN_6_O_3_): C (69.14, 69.01), H (5.29, 5.59), N (14.23, 14.06).

#### 3-((((1-Benzyl-1H-tetrazol-5-yl)methyl)(4-fluorobenzyl)amino)methyl)-6-methoxyquinolin-2(1H)-one (**7**)

Starting from **17a** (70 mg, 0.2 mmol) and **18b** (49 mg, 0.2 mmol) the title compound was prepared following the same procedure of **1**. The crude product was purified by flash chromatography on silica gel (EtOAc) to give **7** as a white solid (60 mg, 52%). ^1^H NMR (300 MHz, CDCl_3_) δ 12.51 (br s, 1H), 7.68 (s, 1H), 7.32–7.22 (m, 3H), 7.18 (d, *J* = 2.7 Hz, 1H), 7.17–7.03 (m, 3H), 6.98 (t, *J* = 8.6 Hz, 2H), 6.93 (d, *J* = 2.6 Hz, 1H), 6.87–6.75 (m, 2H), 5.50 (s, 2H), 3.93 (s, 2H), 3.85 (s, 3H), 3.73 (s, 2H), 3.71 (s, 2H). ^13^C NMR (75 MHz, CDCl_3_) δ 164.1, 163.9, 160.9, 155.6, 152.5, 140.2, 133.8, 133.5, 133.0, 131.3, 131.2, 129.1, 128.9, 128.7, 127.5, 120.6, 120.3, 117.4, 115.8, 115.5, 108.9, 58.3, 56.0, 53.4, 50.9, 46.1; MS (ESI) *m*/*z* 507 [M + Na]^+^; analysis (calcd., found for C_27_H_25_FN_6_O_2_): C (66.93, 67.03), H (5.20, 4.82), N (17.34, 16.97).

#### 3-((((1-Benzyl-1H-tetrazol-5-yl)methyl)(4-fluorobenzyl)amino)methyl)quinolin-2(1H)-one (**8**)

Starting from **17a** (70 mg, 0.2 mmol) and **18c** (40 mg, 0.2 mmol) the title compound was prepared following the procedure described for the preparation of **1**. The crude material was purified by flash chromatography on silica gel (EtOAc) to give **8** as a white solid (50 mg, 46%). ^1^H NMR (300 MHz, CDCl_3_) δ 12.23 (br s, 1H), 7.72 (s, 1H), 7.61–7.46 (m, 2H), 7.38–7.19 (m, 4H), 7.19–7.03 (m, 3H), 6.98 (t, *J* = 8.2 Hz, 2H), 6.81 (d, *J* = 8.0 Hz, 2H), 5.51 (s, 2H), 3.94 (s, 2H), 3.73 (s, 2H), 3.71 (s, 2H). ^13^C NMR (75 MHz, CDCl_3_) δ 164.1, 160.9, 152.5, 140.7, 138.3, 133.8, 133.4, 131.3, 131.2, 131.0, 129.1, 128.7, 128.0, 127.4, 123.2, 119.8, 115.9, 115.8, 115.5, 58.3, 53.5, 50.9, 46.2. MS (ESI) *m*/*z* 477 [M + Na]^+^; analysis (calcd., found for C_26_H_23_FN_6_O): C (68.71, 68.83), H (5.10, 5.36), N (18.49, 18.87).

#### 3-((((1-Benzyl-1H-tetrazol-5-yl)methyl)benzylamino)methyl)-6,7-dimethoxyquinolin-2(1H)-one (**9**)

Starting from **17c** (25.0 mg, 0.1 mmol) the title compound was prepared following the same procedure of **1**. The crude product was purified by flash chromatography on silica gel (33% DCM in CH_3_CN) to give **9** as a white solid (10 mg, 23%). ^1^H NMR (CD_3_OD): δ 7.75 (s, 1H), 7.32–7.27 (m, 5H), 7.25–7.13 (m, 3H), 7.05 (s, 1H), 6.87–6.84 (m, 3H), 5.55 (s, 2H), 3.94 (s, 2H), 3.90 (s, 3H), 3.86 (s, 3H), 3.70 (s, 2H), 3.64 (s, 2H). MS (ESI) *m/z* 519 [M + Na]^+^; analysis (calcd., found for C_28_H_28_N_6_O_3_): C (67.73, 67.55), H (5.68, 5.92), N (16.92, 17.06).

#### 3-((((1-Benzyl-1H-tetrazol-5-yl)methyl)(4-chlorobenzyl)amino)methyl)-6,7-dimethoxyquinolin-2(1H)-one (**10**)

Starting from **17d** (40.0 mg, 0.1 mmol), the title compound was prepared following the same procedure of **1**. The crude product was purified by flash chromatography on silica gel (33% DCM in CH_3_CN) to give **10** as a white solid (30 mg, 44%). ^1^H NMR (CDCl_3_): δ 12.66 (br s, 1H), 7.66 (s, 1H), 7.33–7.20 (m, 4H), 7.16–7.08 (m, 3H), 6.88 (s, 1H), 6.79–6.76 (m, 3H), 5.45 (s, 2H), 3.92 (s, 5H), 3.90 (s, 3H), 3.77 (s, 2H), 3.74 (s, 2H). MS (ESI) *m/z* 553 [M + Na]^+^; analysis (calcd., found for C_28_H_27_ClN_6_O_3_): C (63.33, 63.39), H (5.13, 4.93), N (15.83, 15.58).

#### 3-((((1-Benzyl-1H-tetrazol-5-yl)methyl)(4-bromobenzyl)amino)methyl)-6,7-dimethoxyquinolin-2(1H)-one (**11**)

Starting from **17e** (35.0 mg, 0.1 mmol) the title compound was prepared following the same procedure of **1**. The crude product was purified by flash chromatography on silica gel (33% DCM in CH_3_CN) to give **11** as a white solid (15 mg, 30%). ^1^H NMR (CDCl_3_): δ 12.04 (br s, 1H), 7.67 (s, 1H), 7.43–7.40 (m, 2H), 7.20–7.13 (m, 6H), 6.89 (s, 1H), 6.81–6.79 (m, 2H), 5.47 (s, 2H), 3.99–3.87(m, 8H), 3.82–3.70 (m, 4H). MS (ESI) *m/z* 597 [M + Na]^+^; analysis (calcd., found for C_28_H_27_BrN_6_O_3_): C (58.44, 58.58), H (4.73, 4.96), N (14.60, 14.71).

#### (Benzyltetrazolyl)-N-(adamantylmethyl)methanamine (**19**)

Starting from **16a** (60.0 mg, 0.3 mmol) and adamantine-1-carbaldheyde (48.0 mg, 0.3 mmol) the title compound was prepared following the same procedure of **17a**. The crude product was purified by flash chromatography on silica gel (2% MeOH in CHCl_3_) to give **19** as a colorless oil (50 mg, 70%). ^1^H NMR (CDCl_3_): δ 7.45–7.22 (m, 3H), 7.32–7.45 (m, 2H), 5.70 (s, 2H), 3.90 (s, 2H), 2.18 (s, 2H), 1.94–1.89 (m, 3H), 1.72–1.58(m, 7H), 1.47–1.44 (m, 5H).

#### 3-((((1-Benzyl-1H-tetrazol-5-yl)methyl)(1-adamantyl methyl)amino)methyl)-6,7-dimethoxyquinolin-2(1H)-one (**12**)

Starting from **19** (50 mg, 0.2 mmol) the title compound was prepared following the same procedure of **10**. The crude material was purified by flash chromatography on silica gel (33% DCM in CH_3_CN) to give **12** as a yellow oil, (20 mg, 25%). ^1^H NMR (CDCl_3_): δ 12.06 (br s, 1H), 7. 89 (s, 1H), 7.10–7.08 (d, *J* = 7.0 Hz, 3H), 6.94–6.90 (d, *J* = 12.4 Hz, 3H), 6.79 (s, 1H), 5.62 (s, 2H), 3.99 (s, 3H), 3.96 (s, 3H), 3.94 (s, 2H), 3.78 (s, 2H), 2.39 (s, 2H), 1.94–1.89 (m, 3H), 1.72–1.58 (m, 7H), 1.47–1.44 (m, 5H); analysis (calcd., found for C_32_H_38_N_6_O_3_): C (69.29, 68.93), H (6.91, 6.64)), N (15.15, 14.90).

### Crystallization, data collection and processing

Crystals of *Lm*TXNPx-FF were grown by hanging drop vapor diffusion method at 293 K. *Lm*TXNPx sample was concentrated to about 12 mg/mL in 20 mM Tris-HCl buffer (pH 7.5) in presence of 50 mM DTT. Aliquots (1.0 μL) of protein solution with 50 mM DTT were mixed with an equal volume of the reservoir solution composed of 20–22% (w/v) PEG3350, 100mM Tris-HCl buffer (pH 7.0), 0.2 M NaSO_4_. Crystals of reduced *Lm*TXNPx grew in few days at 293 K and belong to the C222_1_ space group with the following cell dimensions: a = 111.8 Å, b = 226.2 Å, c = 91.7 Å.

The crystals were cryo-protected by adding 20% glycerol (v/v) to the mother liquor, mounted in nylon loops and flash-frozen by quick submersion into liquid N2 for transport to the synchrotron-radiation source. All X-ray diffraction data were collected as 0.2 oscillation frames at 100 K on the beam line XRD1 at ELETTRA (Basovizza (Ts), Italy) using a Pilatus 2M detector. The data were processed and scaled using the program XDS^21^. Crystal parameters, data-collection and refinement statistics are presented in Table 2.

It is worth mentioning that the crystallization conditions used to crystallize the *Lm*TXNPx-LU (PDB code: 3TUE)^11^ were slightly different than those used to crystallize *Lm*TXNPx-FF; the mother liquor solution contained 24–26% PEG 3350, 100 mM Bis Tris propane (PH = 8.0), 50 mM DTT and 0.2 M KSCN. As a matter of fact, *Lm*TXNPx-LU crystallizes in the same space group as *Lm*TXNPx-FF (C222_1_) but with a cell volume 6% smaller (cell dimensions: a = 113 Å, b = 211 Å, c = 91 Å).

### Structures solution and refinement

The structure of *Lm*TXNPx in FF conformation was solved by molecular replacement, performed with the program Molrep^22^ using as search model the pentameric structure of *Lm*TXNPx in LU conformation (PDB code 3TUE)^11^. The molecular replacement produced a clear solution corresponding to a pentamer in the asymmetric unit.

Refinement of the atomic coordinates and displacement parameters were carried out using Refmac5^23^. The structure has been refined at a 2.34 Å resolution to a R_factor_ of 19.6% and a Rfree of 24.7%. Manual fitting and model building were performed using COOT^24^.

The quality of the models was assessed using the program PROCHECK^25^. All refinement statistics are presented in Table 2. Structural figures were generated with PyMOL.

### Surface Plasmon Resonance (SPR) measurements

SPR experiments were carried out using a SensiQ Pioneer system (SensiQ, ICxNomadics Inc.). The interaction of the immobilized protein with the analytes was detected by mass concentration-dependent changes of the refractive index on the sensor chip surface. The changes in the observed SPR signal are expressed as Resonance Units (RU). Typically, a response change of 1000 RU corresponds to a change in the surface concentration on the sensor chip of about 1 ng of protein per mm^2^.

The sensor chips (COOH5 SensiQ) were chemically activated by injection of 250 μl of a 1:1 mixture of *N*-hydroxysuccinimide (50 mM) and *N*-ethyl-*N*-(3-dimethylaminopropyl)carbodiimide (200 mM) at a flow rate of 25 μl/min. Recombinant *Lm*TXNPx was immobilized on the activated sensor chip *via* amine coupling. The reaction was carried out at a rate of 10 mL/min in 20 mM sodium acetate at pH 4.5; the remaining ester groups were blocked by injecting 100 μL of 1 M ethanolamine hydrochloride. Recombinant *Lm*TXNPx was captured to approximately 2000 RU. The analytes were dissolved at a concentration of 10 mM or 20 mM in dimethylsulfoxide (DMSO), and diluted 1:100 in HEPES-buffered saline (HBS: 10 mM HEPES, pH 7.4; 150 mM NaCl; 0.005% surfactant P20).

FastStep injections of samples (100 or 200 μM analytes in HBS + 1% DMSO), and reference buffer (HBS + 1% DMSO) were performed: either the inhibitor and reference buffer were automatically diluted in HBS and injected by 7 serial doubling steps (step contact time = 15 s, nominal flow rate = 200 μl/min). The following analytes were injected: 0–17 s: analyte concentration = 3.12 μM; 17–33 s: 6.25 μM; 33–48 s: 12.5 μM; 48–62 s: 25 μM; 63–78 s: 50 μM; 78–93 s: 100 μM; 94–100 s: 200 μM; for each injection, a maximal RU value was obtained. In control experiments, the sensor chip was treated as described above in the absence of immobilized protein.

Each sensorgram is the average of three different experiments. Sensorgrams were subjected to global analysis using QDat software 2.2.0.23, and for each analyte concentration a % Response was calculated, allowing a local Rmax fit (according to the molecular weight of each compound) and displaying as a response relative to the Rmax. % Response vs. analyte concentration was plotted and a *K*_D_ value was calculated for each analyte by fitting a simple 1:1 steady-state affinity model to the data.

### Inhibition assay

Competition kinetics have been performed for determining the activity of the compounds selected by HTD. The ability of the compounds to inhibit the H_2_O_2_ reduction by *Lm*TXNPx has been determined by competition approach utilizing the well-known reactivity of H_2_O_2_ with horseradish peroxidase (HRP)^16^,^20^,^26^. HRP is a heme-containing peroxidase, which uses hydrogen peroxide (by reducing it to water) to oxidize different organic substrates to free radicals. The reaction takes place in three steps:

1. PFe(III) + H_2_O_2_ → P^·+^Fe(IV) = O + H_2_O
2. P^·+^Fe(IV) = O + X → X^•^ + PFe(IV) = O
3. PFe(IV) = O + X + 2H^+^ → PFe(III) + X^·^ + H_2_O

Where PFe(III) is HRP (the native protein), P^·+^Fe(IV) = O is the HRP compound I (HRP-I), PFe(IV) = O is the HRP compound II (HRP-II) and X is a generic substrate. The formation of HPR-I results in a change in light absorption in the visible range (Δε_403 nm_ = 5.4 × 10^4^ M^−1^cm^−1^). For kinetic studies the HRP oxidation is monitored at 398 nm (Δε_398 nm_ = 4.2 × 10^4^ M^−1^cm^−1^) instead of 403 nm (wavelength corresponding to the maximum of HRP absorbance) in order to avoid interferences arising from HRP-I reduction to HRP-II. This method is usually used to determine the second order rate constant of 2Cys- Peroxiredoxin reaction with hydrogen peroxide and peroxinitrite^16^,^20^,^26^.

Thus, H_2_O_2_-mediated HRP oxidation is a two-electron oxidation process, leading to the formation of HRP-I, followed at 398 nm. The addition of increasing concentrations of pre-reduced *Lm*TXNPx led to a lower yield in HRP-I since two reactions take place in solution:
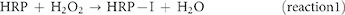

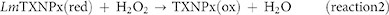


As described by Fiorillo *et al.*^6^ the ΔA can be written as a function of the HRP and *Lm*TXNPx concentrations:
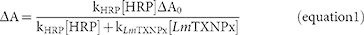
where k_HRP_ and k*_Lm_*_TXNPx_ are the second-order rate constants for the combination of HRP and *Lm*TXNPx with hydrogen peroxide and display the following values: k_HRP_ = 2.0 × 10^7^ M^−1^ s^−1^ k*_Lm_*_TXNPx_ = 3.4 × 10^7^ M^−1^ s^−1^ and ΔA_0_ is the change in absorbance due to the HRP-I formation in the absence of *Lm*TXNPx.

In presence of the inhibitor in addition to the reactions1 and 2 the following equilibrium exists in solution: 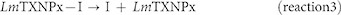


Where I indicates the inhibitor and *Lm*TXNPx-I indicates the complex between *Lm*TXNPx and the inhibitor. The addition of active *Lm*TXNPx inhibitors led to a decrease in the free *Lm*TXNPx and the consequent recover in the HRP-I yield and therefore to a partial or total recover of the ΔA.

Thus, the equation 1 can still be used but [*Lm*TXNPx] should be expressed as a function of inhibitor concentration and the dissociation constant of the *Lm*TXNPx-I complex (which is equal to the inhibition constant):
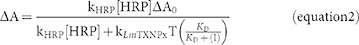


Where T is the *Lm*TXNPx concentration in the absence of the inhibitor, [I] is the concentration of the inhibitor in the free form, and *K*_D_ is the dissociation/inhibition constant.

The HRP-based indirect kinetic assays to test the inhibitors was performed in the following way: HRP 2 μM was exposed to H_2_O_2_ 0.25 μM in sodium phosphate buffer pH 7.4 and 25°C, either in the absence or in the presence of the indicated concentrations of reduced *Lm*TXNPx and in the absence or in the presence of inhibitors. The relative changes in absorbance at 398 nm (ΔA/ΔA_0_), reflecting HRP-I formation, were determined.

The equation 2 was arranged as follow to tentatively fit the data:
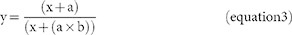
where: y = ΔA/ΔA_0_, x = [I], a = *K*_D_ and b = (1 + (k*_Lm_*_TXNPx_T/k_HRP_C_HRP_).

Many attempts to fit the experimental data have been done but failed. The reasons are reported below:

- High inhibitor concentrations (>10 μM). When the concentration of the inhibitors is high, non-specific interaction with both HRP-I and HRP-II may take place thereby affecting the spectroscopic signal;
- Low inhibitor concentrations (<10 μM). The equations 2 and 3 are effective only when the [I] (concentration of inhibitor in the free form) ≈[I]_tot_, i.e. when [I]_tot_ ≫ [HRP] + [TXNPx]. Thus, at a concentration <10 μM which is ≤ [HRP] + [TXNPx], the use of equations 2 and 3 yields big errors.

The compounds ability to inhibit TXNPx has been evaluated by simply measuring the following ratio: ΔA/ΔA_0_ where ΔA_0_ is the difference in absorbance between HRP and compound I (ΔA_0_ = 0.04) and ΔA is the difference in absorbance between HRP and compound I in the presence of *Lm*TXNPx and 100 μM inhibitors. All the compounds have been previously tested for their ability to inhibit HRP at a concentration of 100 μM.

### Computational Details

#### a) Protein preparation and binding sites analysis

The three-dimensional structures of *Lm*TXNPx in LU and FF conformation (PDB IDs: 4K1F and 3TUE, respectively) downloaded from Protein Data Bank were refined by means of Maestro suite applying protein preparation wizard protocol. The structures were amended according to Figure 2C. The refinement protocol was employed in order to get optimized structures for performing docking studies and/or MD simulation. The refinement of the models was conducted in three subsequent steps: i) addition of hydrogens, ii) optimization of the orientation of OH groups, of asparagine and glutamine, and the protonation of histidine, iii) refinement of the model by means of impref application applying a RMSD of 0.30. This application performs a series of cycles of energy minimization adopting MM engine method, using OPLS_2005 as force field^27^. The same procedure was applied for the **1**-*Lm*TXNPx complex before submitting it to MD simulation according to Desmond guidelines (Desmond User manual version 3.0). The binding sites analysis was performed by using SiteMap application (SiteMap, version 2.5, Schrödinger, LLC, New York, NY, 2011) with default settings. The maps were saved as .ccp4 files and visualized in PyMOL.

#### b) 3D-chemical databases preparation

Our proprietary database was built in Maestro suite. The structures were minimized by means of MacroModel using the OPLS-AA_2005 as force field. GB/SA model was used in order to simulate the solvent effect applying “no cutoff” for non-bonded interactions. PRCG method (1000 maximum iterations and 0.001 gradient convergence threshold) was employed. Compounds were then submitted to LigPrep program (version 2.5, Schrödinger, LLC, New York, NY, 2011), producing a possible ionization state taking also into account all enantiomers and tautomers at cellular pH value (7.0 ± 0.5). The resulting output was saved as .sdf file. Asinex 3D-chemical database was downloaded by ZINC website (https://zinc.docking.org/) as 25 separate .sdf files.

#### c) HTD protocol

High-Throughput Docking was performed by means of GOLD 5.0.2 program from CCDC, UK using the Genetic algorithm (GA)^15^. This technique permits a partial flexibility of receptor and full flexibility of compounds. 125000 GA operations were executed for each of the 50 independent GA runs. A search efficiency of 100% was employed. For docking calculation, the active site were defined through XYZ coordinates by adopting a radius of 8 Å from the Arg128 (representing roughly the center of the binding site). Default setting for H-bonds (2.5 Å) and for van der Waals interactions (4.0 Å) was applied. GoldScore scoring function was employed for ranking the docked compounds. Compounds shown in Table S1 were purchased from Asinex.

#### d) Molecular Dynamics simulation

According to Desmond guidelines, after the above-mentioned preparation, the *Lm*TXNPx and the **1**-*Lm*TXNPx complex were submitted to MD simulation. These calculations were performed by means of Desmond 3.0 package *via* maestro GUI. We have used an orthorhombic box full of water molecules represented by TIP3P model^28^. OPLS_2005 force field^27^ was adopted for the calculations. Ions (Na^+^ and Cl^−^) were implemented in the box in order to obtain a resulting salt concentration of 0.15 M that represents the biological concentration of monovalent ions. A constant temperature of 300 K and a pressure of 1.01325 bar were used, employing NPT as ensemble class. For integrating the equations of motion, RESPA^29^ integrator was applied with 2 fs as inner time step. Constant temperature and pressure were maintained during the simulations employing Nose–Hoover thermostats^30^ and the Martyna–Tobias–Klein method^31^. PME technique^32^ was used to calculate long range electrostatic contacts. 9 Å was used as cut-off for van der Waals and short-range electrostatic contacts. Systems were equilibrated by default parameters with subsequent controlled minimizations and MD simulations applied to slowly relax the selected systems. Consequently, a single trajectory for *Lm*TXNPx of 20 ns was calculated. Concerning **1**-*Lm*TXNPx complex the MD simulation (20 ns) was performed in duplicate in order to improve the reliability of the presented model of interaction. The obtained trajectory files were examined by using both tools (Quality Analysis and Simulation Event Analysis Simulation) provided in Desmond. The above-mentioned tools were employed to produce all plots regarding MD simulations reported in this paper.

#### e) Estimated free-binding energies

Prime/MM-GBSA technique provided in Prime software^33^ consists in computing the variation between the free and the complex state of both the ligand and the protein after energy minimization. The method was applied on the docking complexes of the ligands developed in the present work. The program was used to assess the free-binding energy (ΔG_bind_) as formerly described by us^17^,^19^,^34^,^35^.

## Author Contributions

M.B., S.G., N.R., A.V. and S.B. designed the synthetic strategy, synthesized and analyzed the compounds. Si.Br. and E.N. performed molecular modelling studies. G.C., G.A. and F.D. purified the protein and performed the SPR experiments and enzyme assays. A.I., A.F. and F.D. solved the X-ray structure and performed structural analysis. S.G., G.C. and A.I. wrote the manuscript. All authors reviewed the manuscript.

## Supplementary Material

Supplementary Information Structure-based discovery of the first non-covalent inhibitors of Leishmania major tryparedoxin peroxidase by high throughput docking.

## Figures and Tables

**Figure 1:**
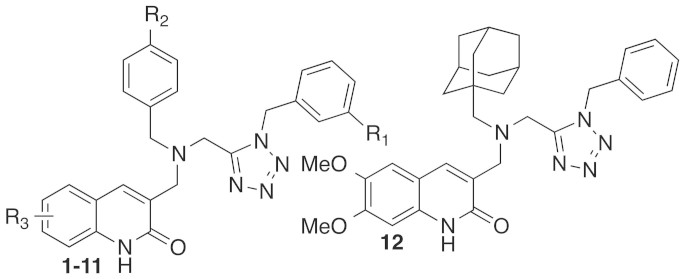
*Lm*TXNPX inhibitors identified in this study (R_1_–R_3_ as defined in Table 1).

**Table 1:** Inhibitors Structure*, Lm*TXNPx Inhibiton Assay and *K*_D_s (μM) Calculated by Surface Plasmon Resonance

Cpds	R_1_	R_2_	R_3_	HRP inhib.	(ΔA/ΔA_0_) × 100^a^	*K*_D_ (μM)
**1**	H	F	6,7-diOMe	No	52 ± 10%	290 ± 13
**2**	NO_2_	F	6,7-diOMe	No	37 ± 9%	172 ± 6
**3**	NH_2_	F	6,7-diOMe	No	44 ± 6%	354 ± 12
**4**	NMe_2_	F	6,7-diOMe	Nd	Nd	156 ± 5
**5**	I	F	6,7-diOMe	No	71 ± 6%	281 ± 11
**6**	Ph	F	6,7-diOMe	No	75 ± 9%	52 ± 1
**7**	H	F	6-OMe	No	60 ± 7%	47 ± 1
**8**	H	F	H	No	45 ± 15%	104 ± 4
**9**	H	H	6,7-diOMe	Yes	-	74 ± 3
**10**	H	Cl	6,7-diOMe	Yes	-	63 ± 3
**11**	H	Br	6,7-diOMe	No	37 ± 20%	112 ± 4
**12**	-	-	-	No	93 ± 6%	39 ± 1

^a^HRP 2 μM was exposed to H_2_O_2_ 0.25 μM in sodium phosphate buffer pH 7.4 and 25°C, in the presence of 2.5 μM *Lm*TXNPx and 100 μM inhibitors. The residual activity of HRP was calculated as ΔA/ΔA_0_ where ΔA_0_ is the difference in absorbance between HRP and HRP-I (ΔA_0_ = 0.04 ± 0.01) and ΔA is the difference in absorbance between HRP and HRP-I in the presence of *Lm*TXNPx and inhibitors.

**Table 2:** Crystal parameters, data collection statistics and refinement statistics of LmTXNPx

Space group	C2221
**Cell dimensions**	
a(Å)	111.811
b(Å)	226.203
c(Å)	91.719
**Data reduction**	
Unique reflections	49113(7500)
Resolution shells (Å)	2.34–50 (2.34–2.48)
Completness	98.9% (94.7%)
Rmerge	0.162(1.07)
I/σ(I)	10.26(1.65)
redundancy	5.48(5.48)
CC(1/2)	99.3(71.2)
**Refinement**	
Rvalue (%)	19.6(22.10)
Rfree (%)	24.7(27.6)
Rms bond lengths(Å)	0.015
Rms bond angle (°)	1.53
Ramachandran Plot analysis	
Residues in the most favored region (%)	97
Residues in allowed region (%)	3
