# Supplementary material for: Structure-based discovery of the first non-covalent inhibitors of Leishmania major tryparedoxin peroxidase by high throughput docking
Source: Sci Rep. 2015 May 7;5:9705. doi: 10.1038/srep09705 (PMC4423475; doi:10.1038/srep09705)
Supplement: Supplementary Information — Structure-based discovery of the first non-covalent inhibitors of Leishmania major tryparedoxin peroxidase by high throughput docking. [file srep09705-s1.doc]

**SUPPORTING INFORMATION**

**Structure-based discovery of the first non-covalent inhibitors of *Leishmania major* tryparedoxin peroxidase by high throughput docking.**

Margherita Brindisi,^‡,†,§^ Simone Brogi,^‡,†,§^ Nicola Relitti,^†,§,∫^ Alessandra Vallone,^†,§^ Stefania Butini,^†,§^ Sandra Gemma,^†,§^,* Ettore Novellino,^†,∫^ Gianni Colotti,^¶^ Gabriella Angiulli,^√^ Francesco Di Chiaro,^√^ Annarita Fiorillo,^√^ Andrea Ilari,^¶^,* Giuseppe Campiani^†,§^

*^†^European Research Centre for Drug Discovery and Development (NatSynDrug), and ^§^Dip. di Biotecnologie, Chimica e Farmacia, University of Siena, via Aldo Moro 2, 53100, Siena, Italy; ^∫^Dip. di Farmacia, University of Naples Federico II, Via D. Montesano 49, 80131 Naples, Italy; ^¶^Istituto Pasteur Fondazione Cenci-Bolognetti and Istituto di Biologia, e Patologia Molecolari IBPM– CNR, c/o Dipartimento di Scienze Biochimiche, Sapienza Università di Roma Piazzale A. Moro, 5, 00185 Roma (Italy); ^√^Istituto di Biologia, Medicina Molecolare e Nanobiotecnologie IBMN–CNR, c/o Dipartimento di Scienze Biochimiche, Sapienza Università di Roma Piazzale A. Moro, 5, 00185 Roma (Italy) and Dipartimento di Scienze Biochimiche, Sapienza Università di Roma Piazzale A. Moro, 5, 00185 Roma (Italy)*

**Scheme S1** S2

**Figure S1** S2

**Figure S2** S2

**Figure S3** S3

**Figure S4** S3

**Figure S5.** S4

**Figure S6**. S4

**Figure S7** S5

**Figure S8** S6

**Figure S9** S6

**Table S1** S7

**Table S2** S9

**Table S3** S9

**Experimental procedures** S10

**Scheme S1**


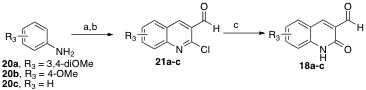


Reagents and conditions: a) Ac_2_O, H_2_SO_4_, 25 °C, 15 min, ref. 1; b) POCl_3_, dry DMF, 85 °C, 12 h; c) AcOH, 110 °C, 12 h.


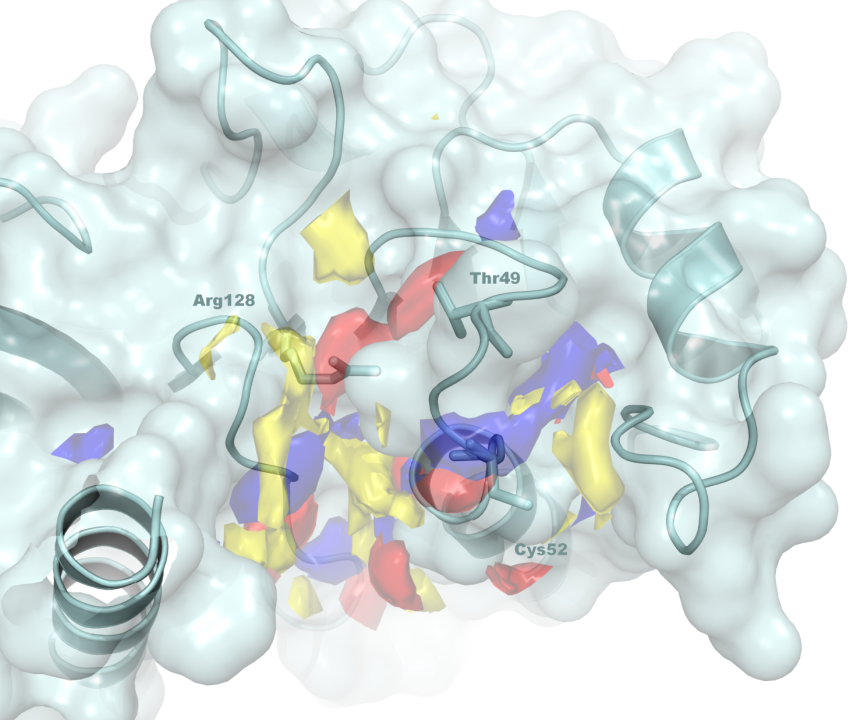


**Figure S1.** Sitemap output of *Lm*TXNPx in LU conformation (volume of the binding site = 217.52 Å^3^).


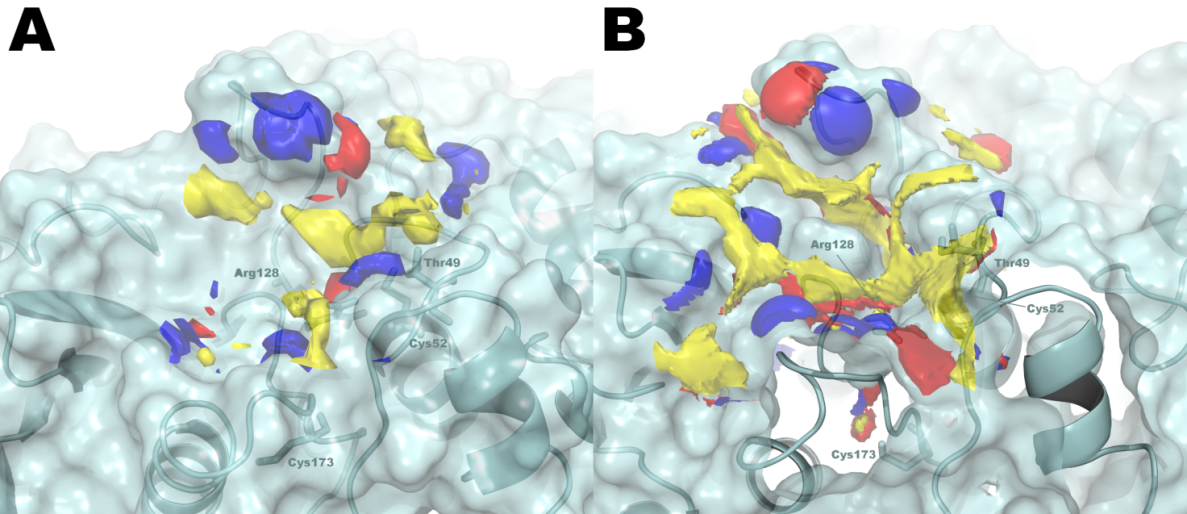


**Figure S2** Binding site enlargement during MD simulation: (A) Sitemap output of *Lm*TXNPx before MD simulation (volume of the binding site = 88.39 Å^3^). (B) Sitemap output of *Lm*TXNPx after 20ns of MD simulation (volume of the binding site 233.37 Å^3^). The maps of binding site for a potential ligand interaction are represented as solid surfaces (red = acceptor; blue = donor; yellow = hydrophobic). Pictures were generated by PyMOL.


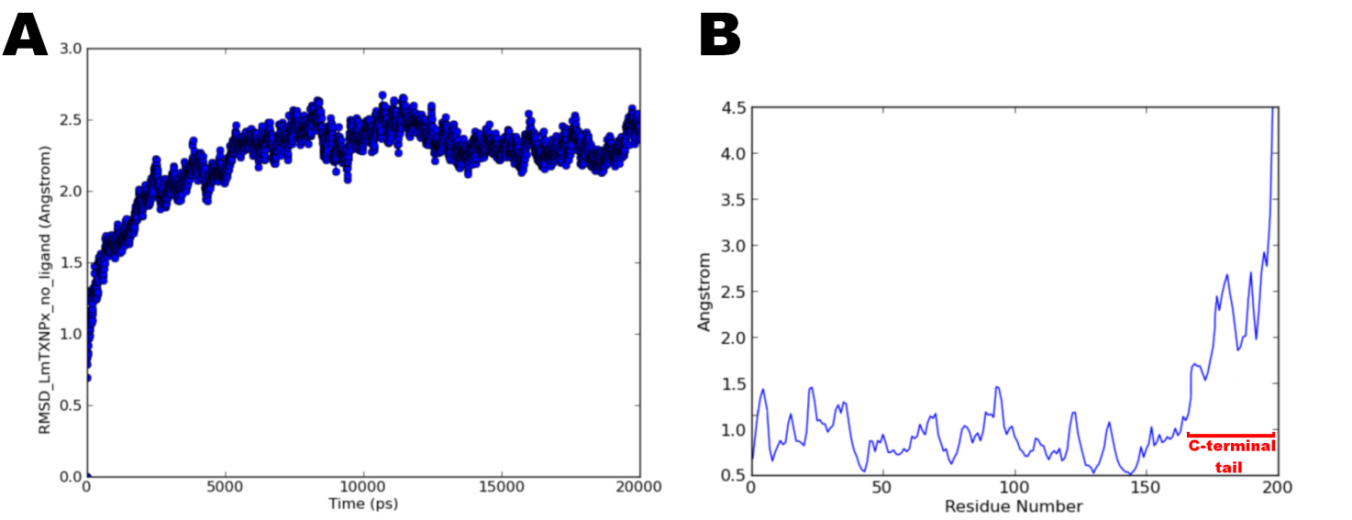


**Figure S3** (A) RMSD (Root Mean Square Deviation) of *Lm*TXNPx. RMSD were calculated between the final conformation and the starting conformation through the 20 ns of the MD simulation. (B) RMSF (Root Mean Square Fluctuation) of all residues of *Lm*TXNPx. The pictures were generated by Simulation Event Analysis implemented in Desmond.


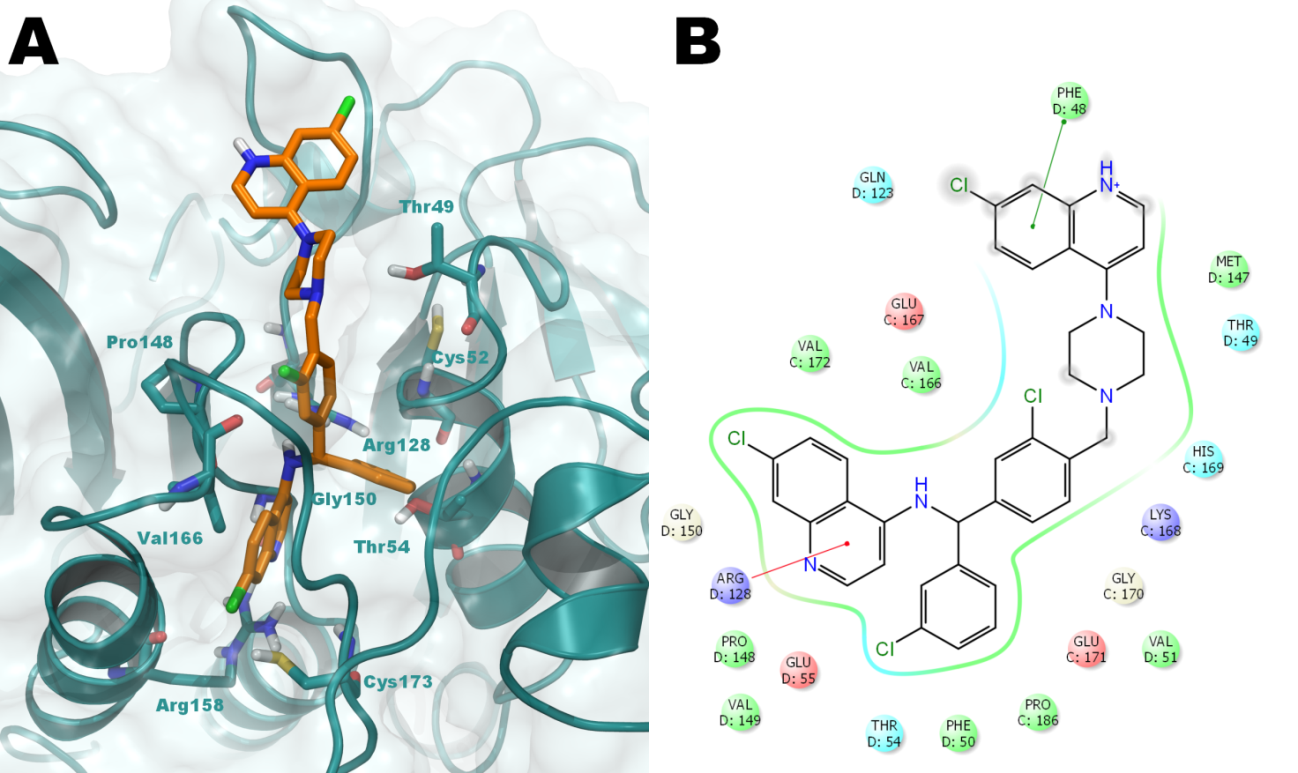


**Figure S4** (A) Putative binding mode of **NF1442** (orange sticks) obtained by GOLD software (GoldScore value 74.61) into predicted active site of the *Lm*TXNPx enzyme (deep teal cartoon). The residues in the binding site of the enzyme are represented by stick. The picture was generated by means of PyMOL (The PyMOL Molecular Graphics System, version 1.6-alpha, Schrödinger, LLC, New York, 2013) (B) Schematic representation of the interactions based on docking calculation. The π-π or cation-π stacking are reported as green and red lines, respectively. The picture was generated by means of the ligand interaction diagram available in Maestro modeling environment suite (Maestro, version 9.2, Schrödinger, LLC, New York, NY, 2011).


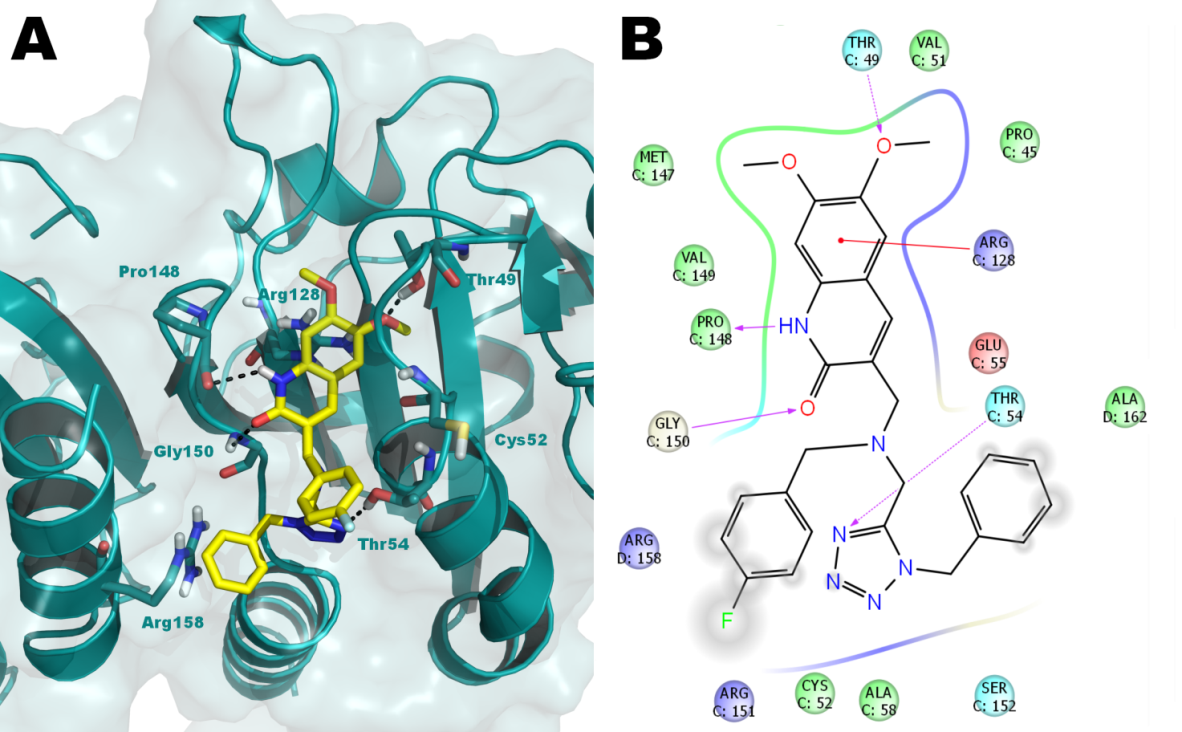


**Figure S5**. Putative binding mode of **1** (yellow sticks) obtained by GOLD software (GoldScore value 67.87) into predicted active site of the *Lm*TXNPx enzyme (deep teal cartoon). The residues in the binding site of the enzyme are represented by stick. H-bonds are reported by grey dotted lines. The picture was generated by means of PyMOL (The PyMOL Molecular Graphics System, version 1.6-alpha, Schrödinger, LLC, New York, 2013) (B) Schematic representation of the interactions based on docking calculation. H-bonds are reported as purple dotted lines while the π-π or cation-π stacking are reported as green and red lines, respectively. The picture was generated by means of the ligand interaction diagram available in Maestro modeling environment suite (Maestro, version 9.2, Schrödinger, LLC, New York, NY, 2011).


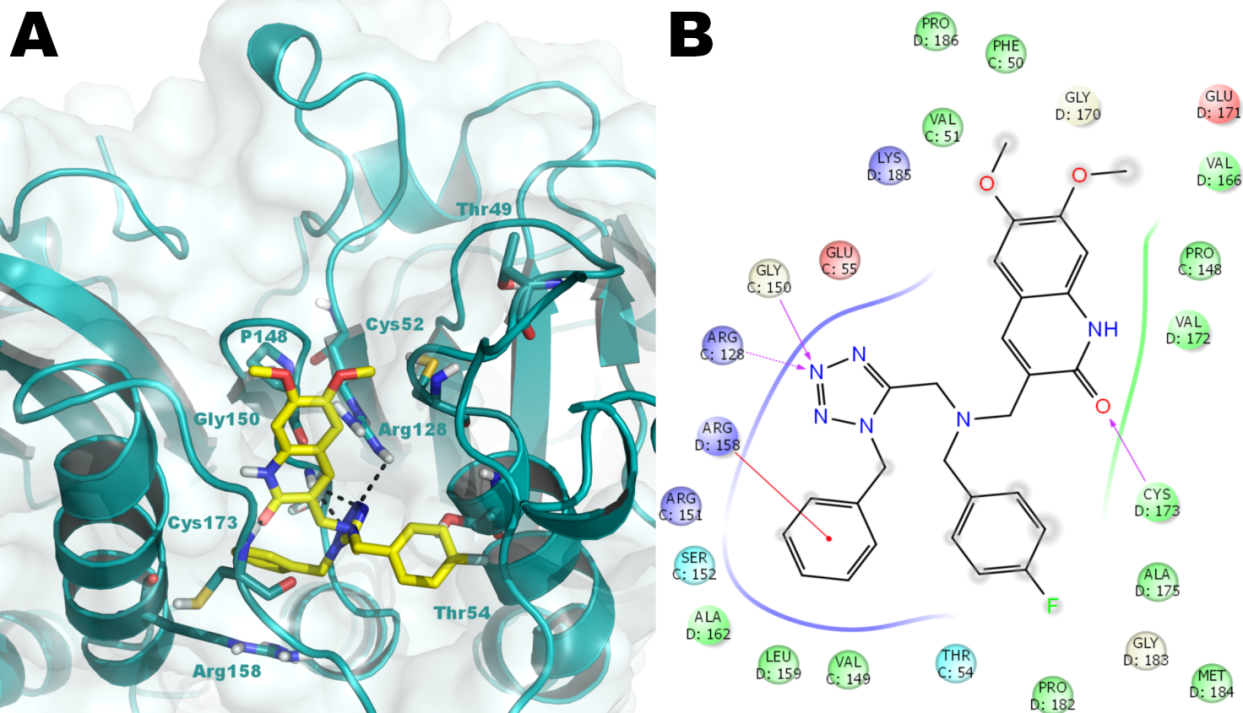


**Figure S6** Snapshot of MD simulation of the complex **1**-TXNPx. The ligand is represented by yellow sticks, while the enzyme is represented by deep teal cartoon. The residues in the binding site of the enzyme are represented by stick. H-bonds are reported by grey dotted lines. The picture was generated by means of PyMOL (The PyMOL Molecular Graphics System, version 1.6-alpha, Schrödinger, LLC, New York, 2013).

**A**

**B**
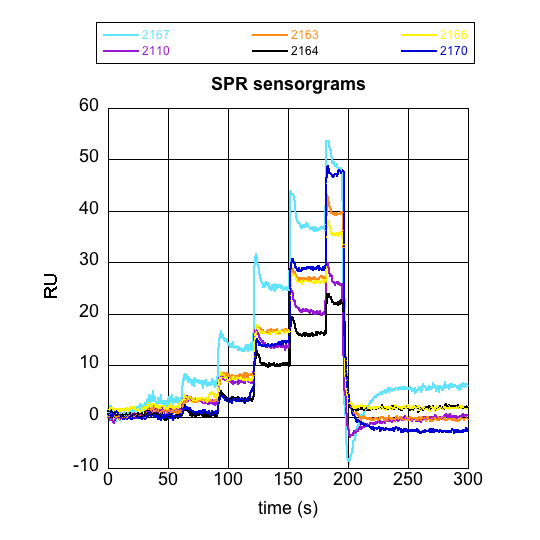


**Figure S7.** SPR experiment: TXNPx was immobilized onto a COOH5 sensorchip; (A): % Response vs. analyte concentration is shown for each analyte, based on two series of SPR experiments. (B) Sensorgrams showing the binding of inhibitors to immobilized TXNPx. The association phase (0-196 s) shows the injection of 6 different inhibitors in HBS buffer + 1% DMSO (10 mM HEPES, pH 7.4; 150 mM NaCl; 0.005% surfactant P20): 0-30 s: 3.12 μM inhibitors; 31-60 s: 6.25 μM inhibitors; 61-90 s: 12.5 μM inhibitors; 91-120 s: 25 μM inhibitors; 121-150 s: 50 μM inhibitors; 151-180 s: 100 μM inhibitors; 181-196 s: 200 μM inhibitors. A single dissociation phase was measured by injecting HBS + 1% DMSO at a rate of 30 µl/min, starting at 196 s. NF2005 =compound 1; NF2163=compound 2 ; NF2164=compound 3; NF2166=compound 4; NF2165=compound 5; NF2168=compound 6; NF2169=compound 7; NF2170=compound 8; NF2109=compound 9; NF2108=compound 10; NF2107=compound 11; NF2167=compound 12;


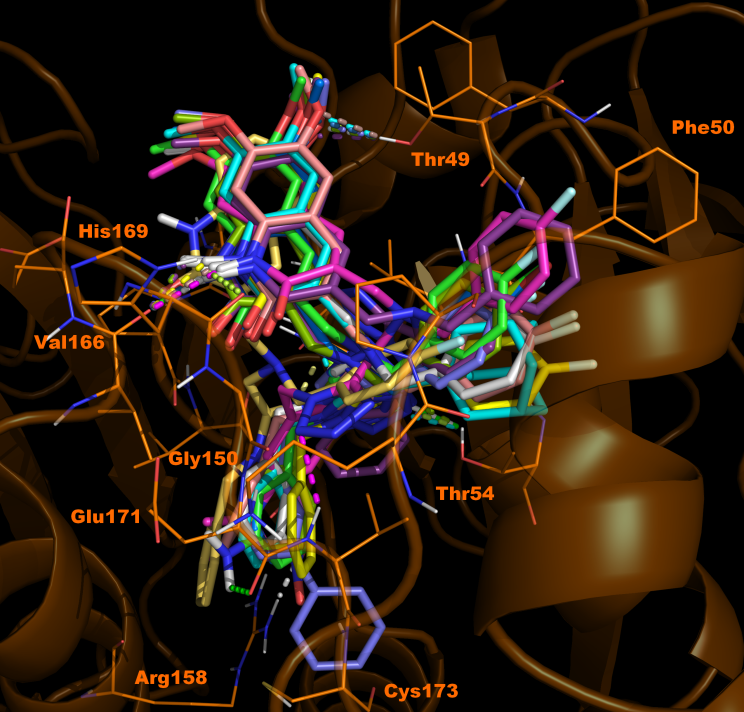


**Figure S8.** Docked poses of all the compounds presented in this study. The picture was generated by means of PyMOL.


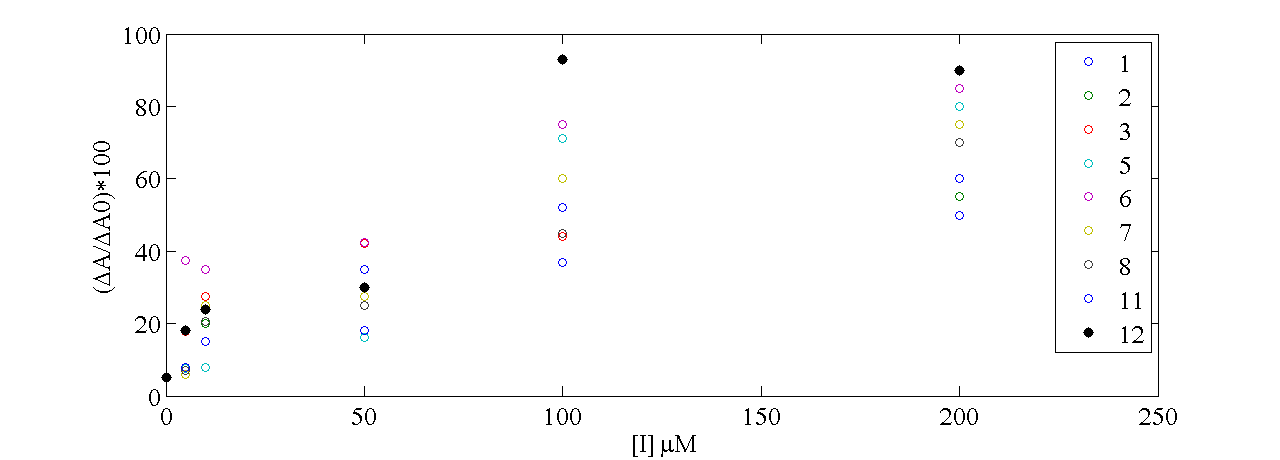


**Figure S9.** Concentration dependence of inhibition (compounds 1-12). HRP 2 µM was exposed to H_2_O_2_ 0.25 µM in sodium phosphate buffer pH 7.4 and 25 °C, in the presence of 2.5 µM *Lm*TXNPx and inhibitors at the following concentrations: 0 µM, 5 µM, 10 µM, 50 µM, 100 µM, 200 µM. The residual activity of HRP was calculated as ΔA/ΔA_0_ where ΔA_0_ is the difference in absorbance between HRP and HRP-I (ΔA_0_ =0.04±0.01) and ΔA is the difference in absorbance between HRP and HRP-I in the presence of *Lm*TXNPx and inhibitors.

**Table S1.** LmTXNPx inhibiton assay first series of compounds.*^a^*

| Compound | Structure | HRP inhibition | (ΔA/ΔA_0_)×100 | Reference |
| --- | --- | --- | --- | --- |
| NF530 | 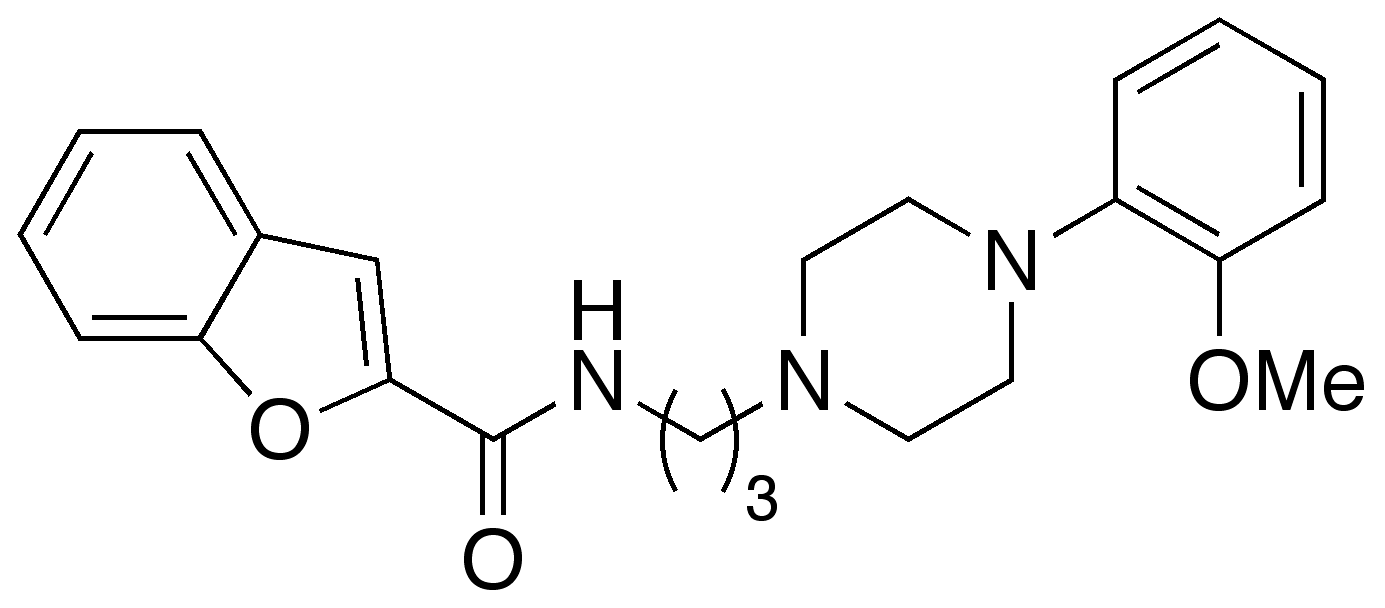 | No | 12 ± 6% | 2 |
| NF843 | 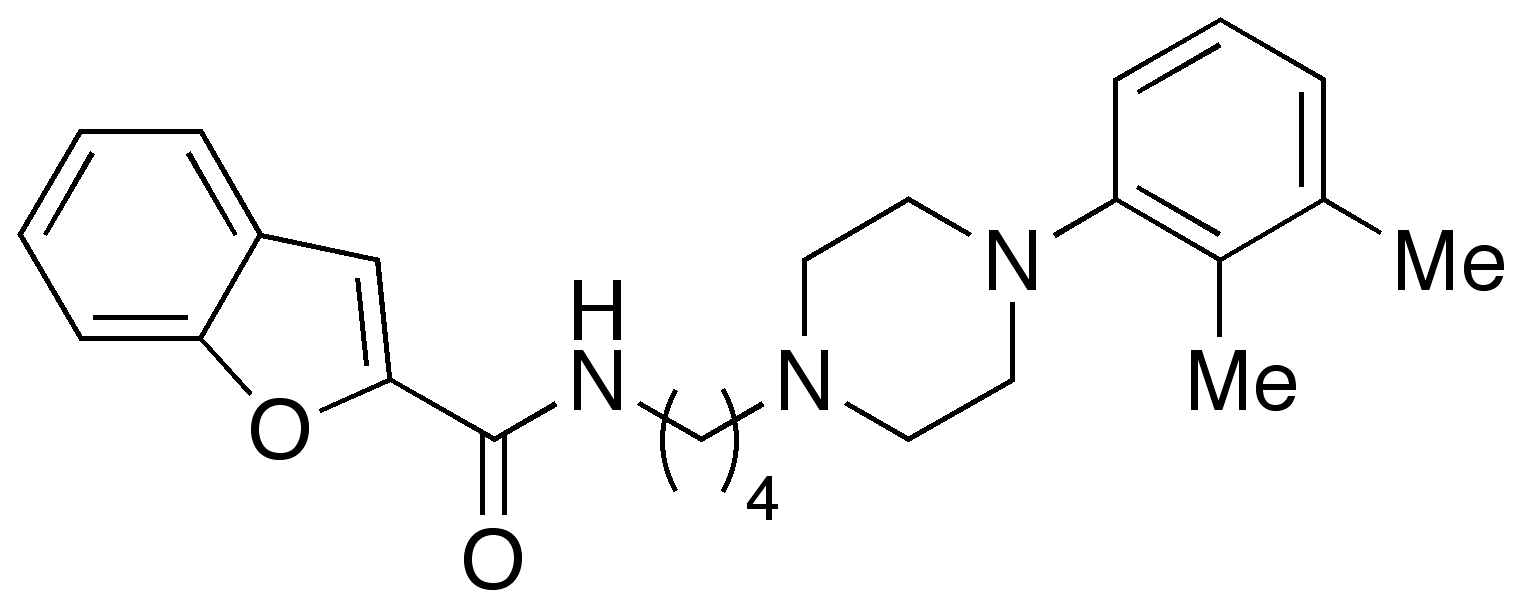 | No | 10 ± 9% | 3 |
| NF844 | 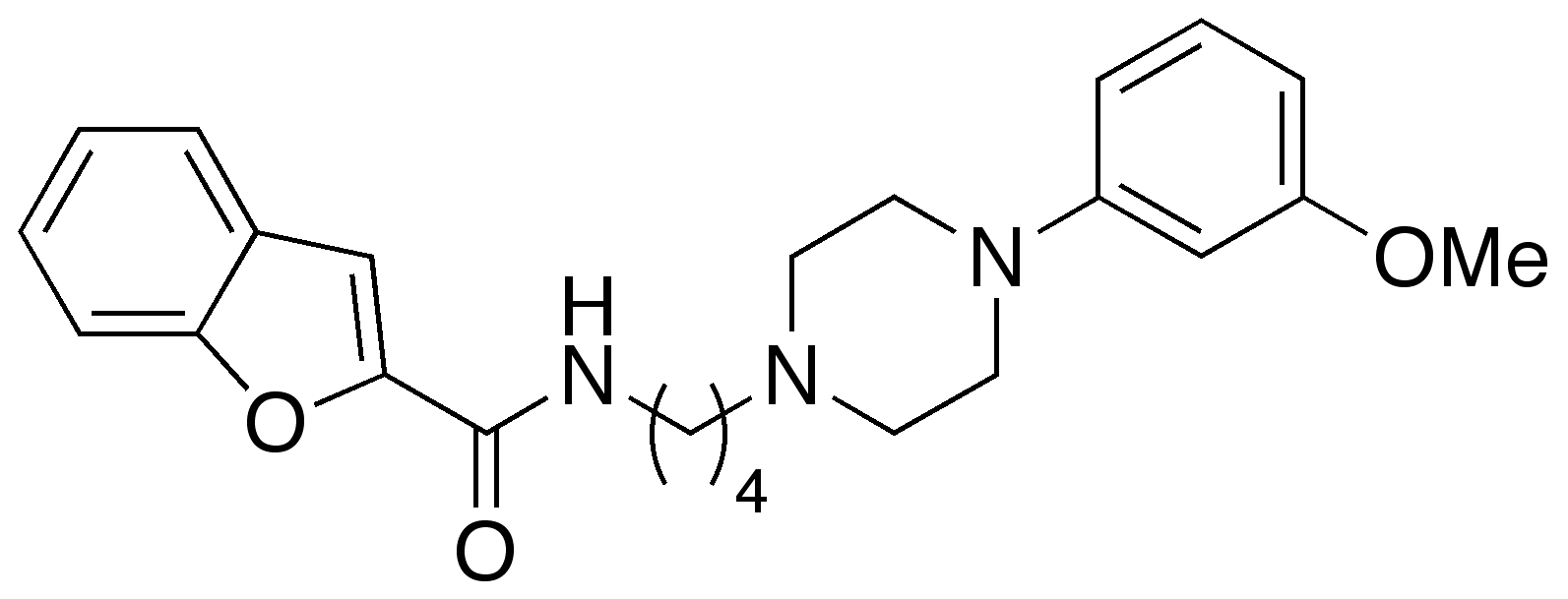 | No | 11 ± 7% | 3 |
| NF864 | 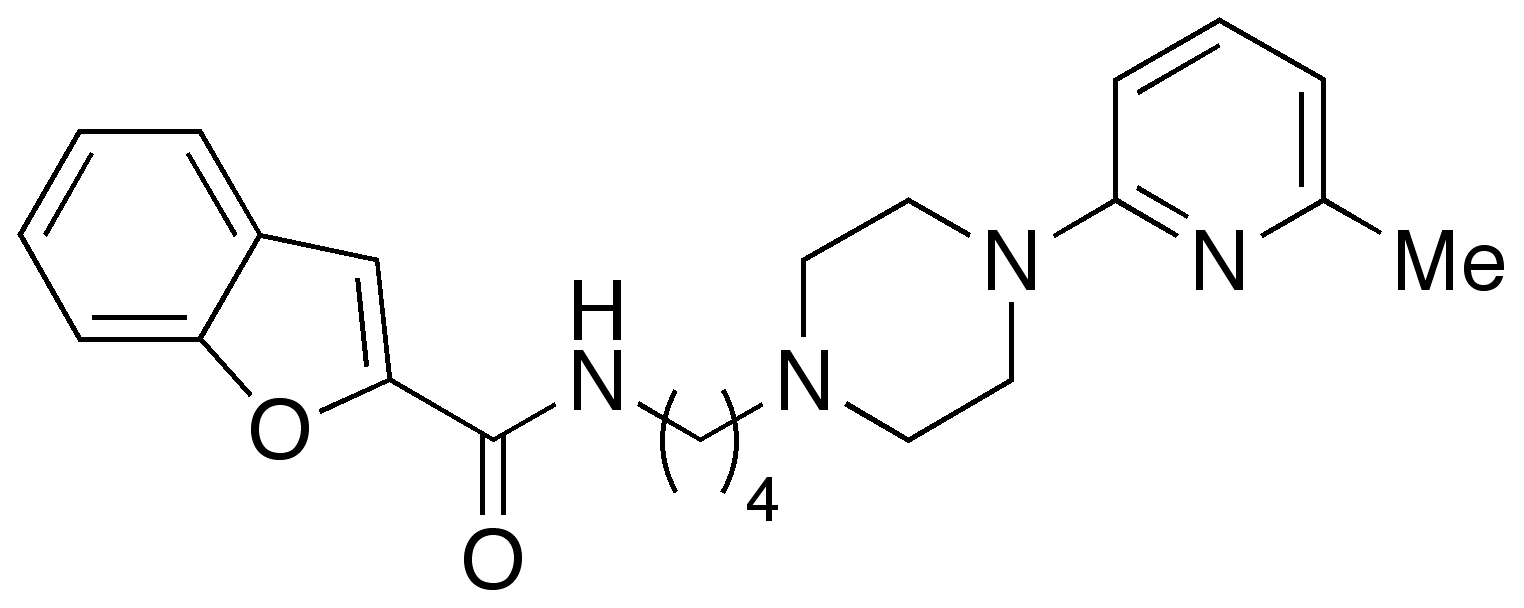 | No | 16 ± 6% | 3 |
| NF877 | 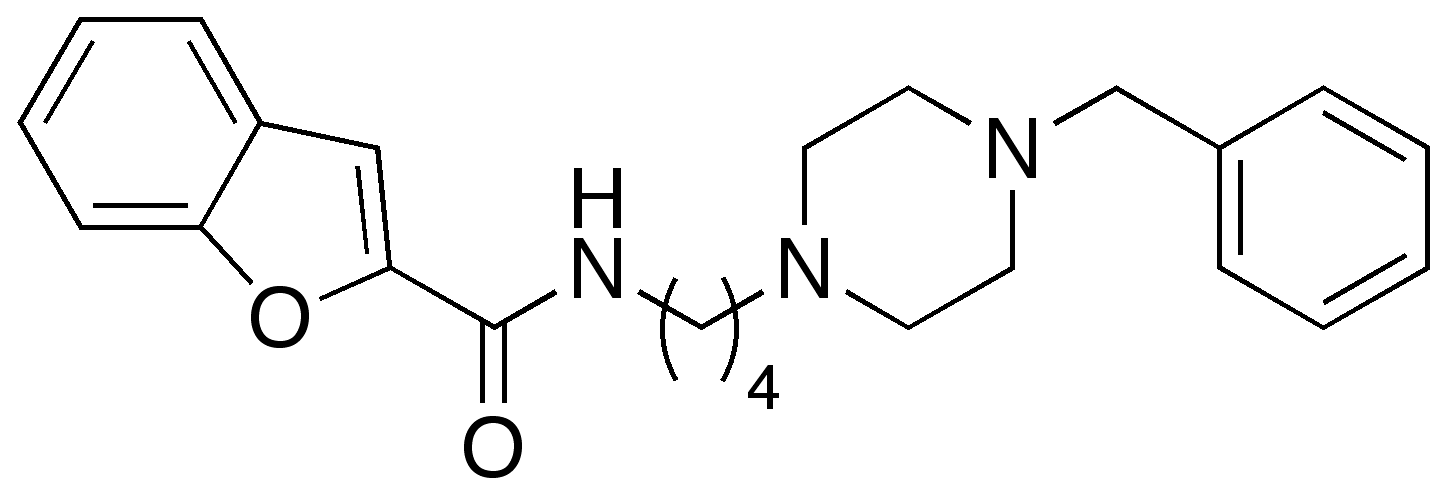 | No | 12 ± 6% | 3 |
| NF1442 | 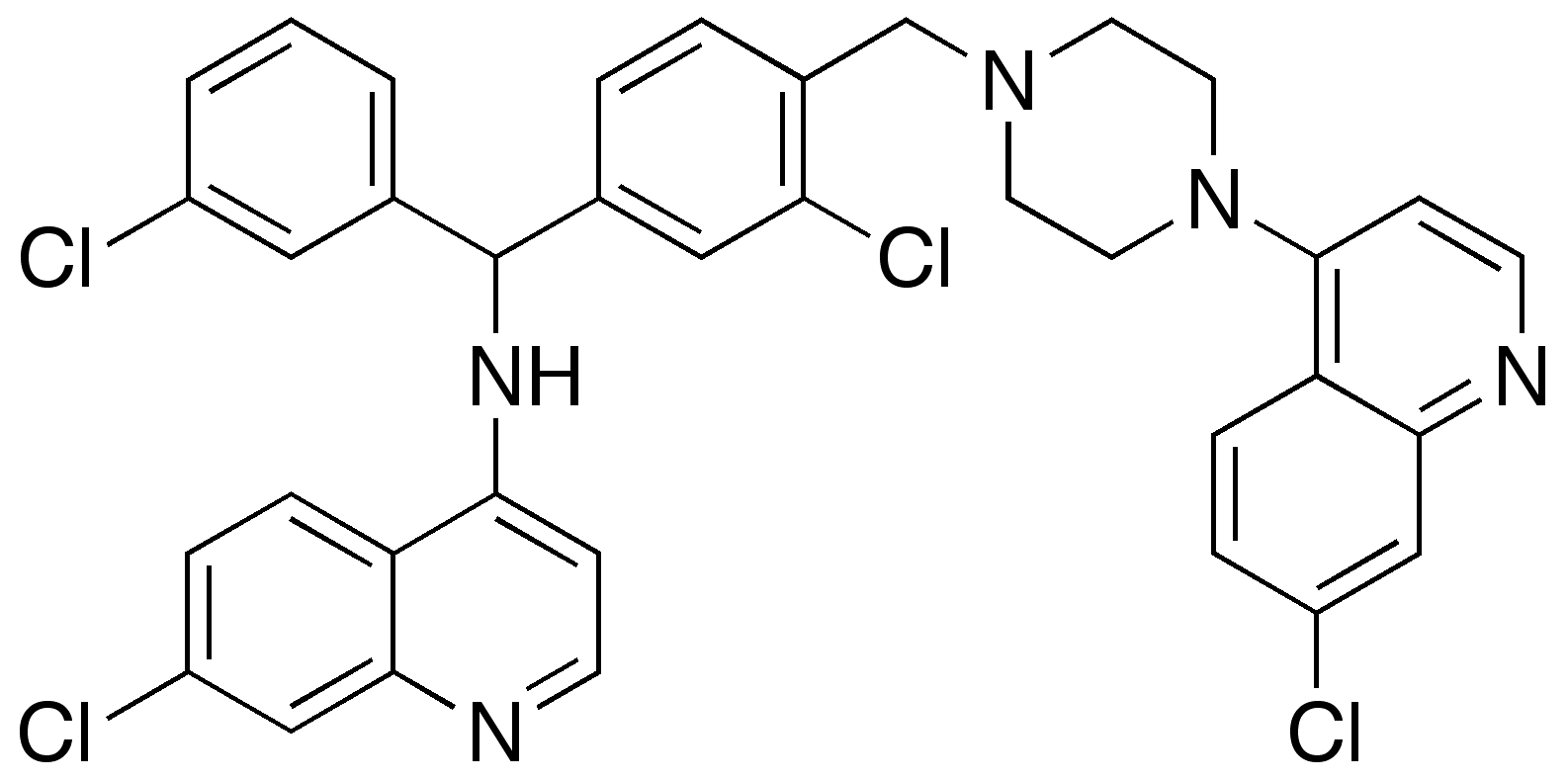 | No | 35 ± 8% | 4 |
| NF1885 | 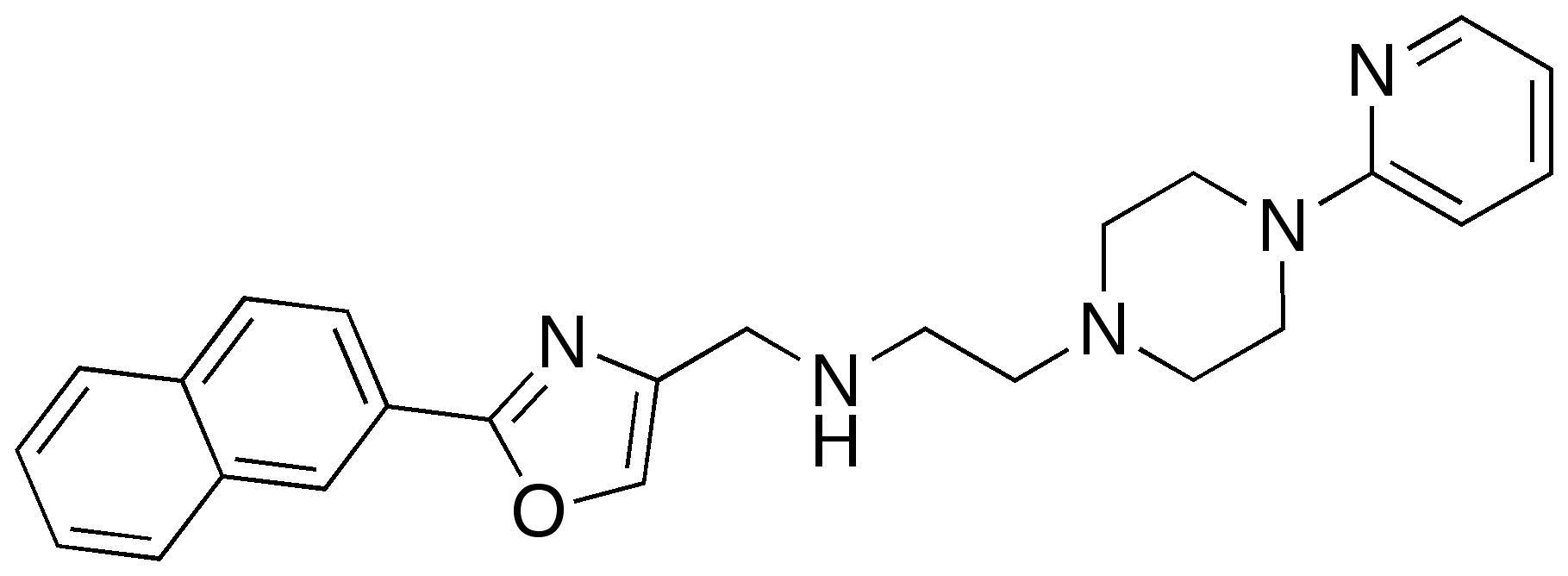 | No | 11 ± 4% | 5 |
| NF1886 | 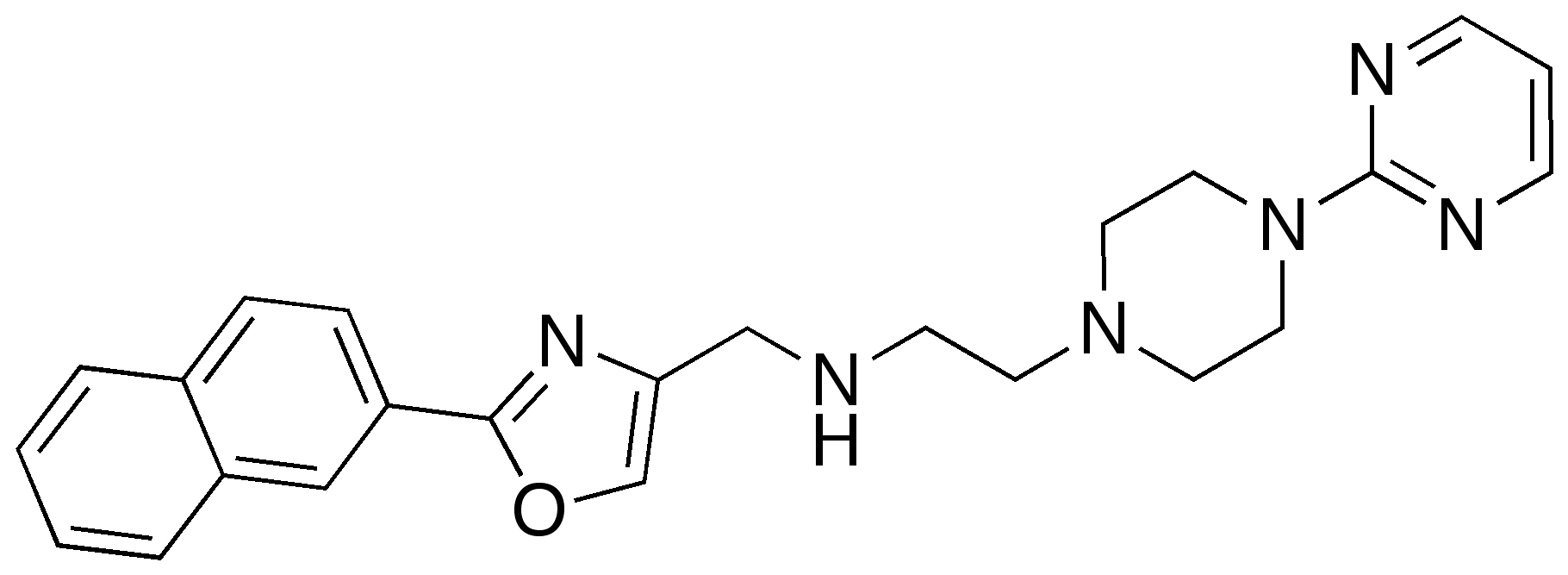 | No | 14 ± 4% | 5 |
| NF2004 | 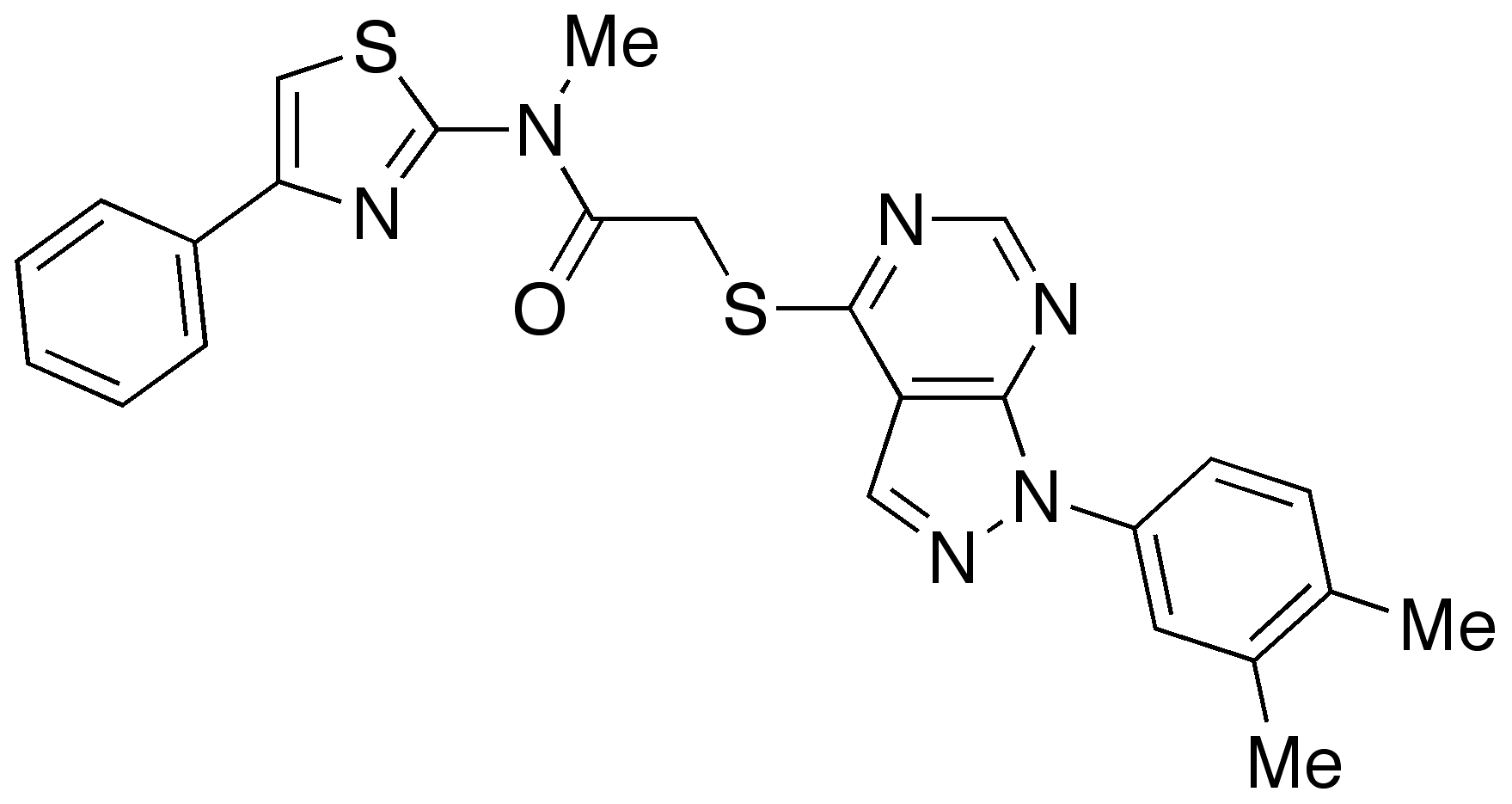 | No | Not soluble | ASN05100142 |
| NF2005 (**1**) | 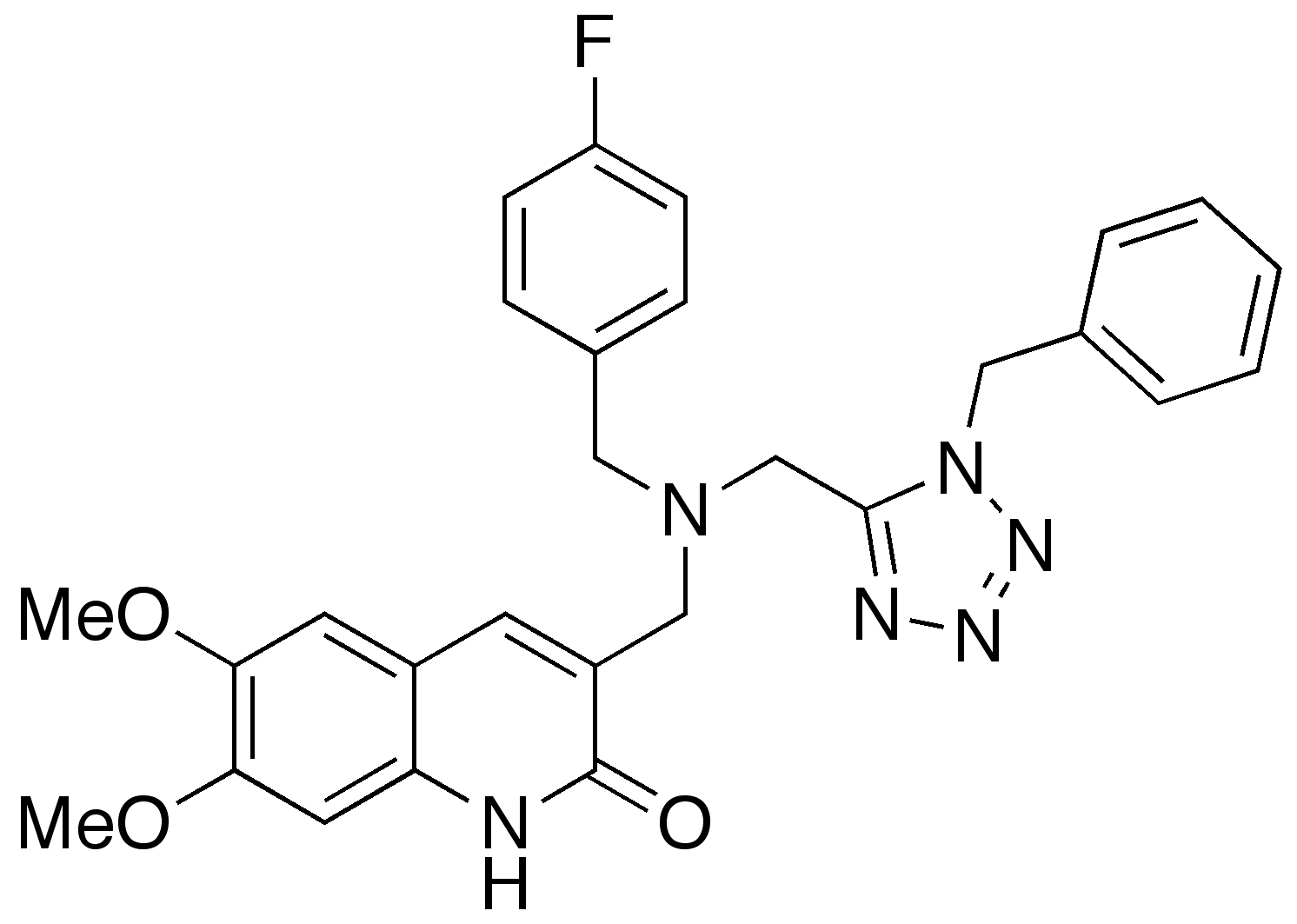 | No | 47 ± 6% | ASN05298733 |
| NF2006 | 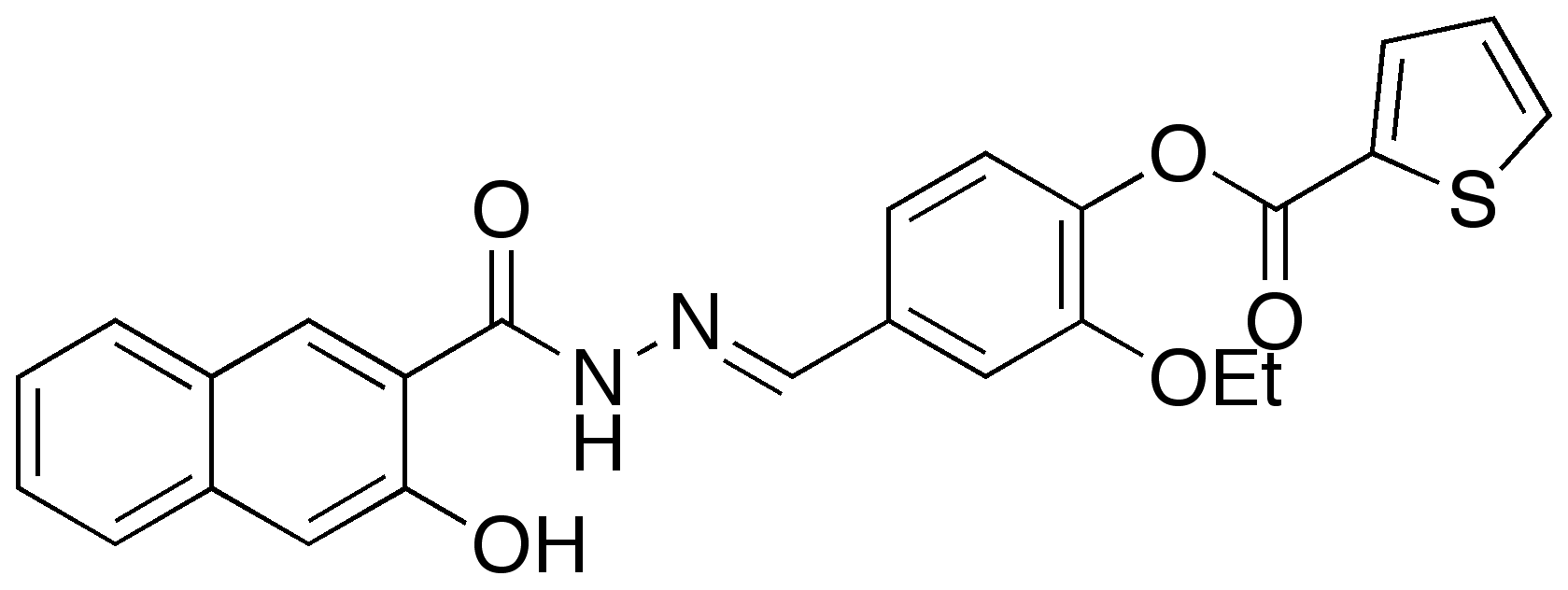 | No | 22 ± 8% | BAS01232110 |
| NF2007 | 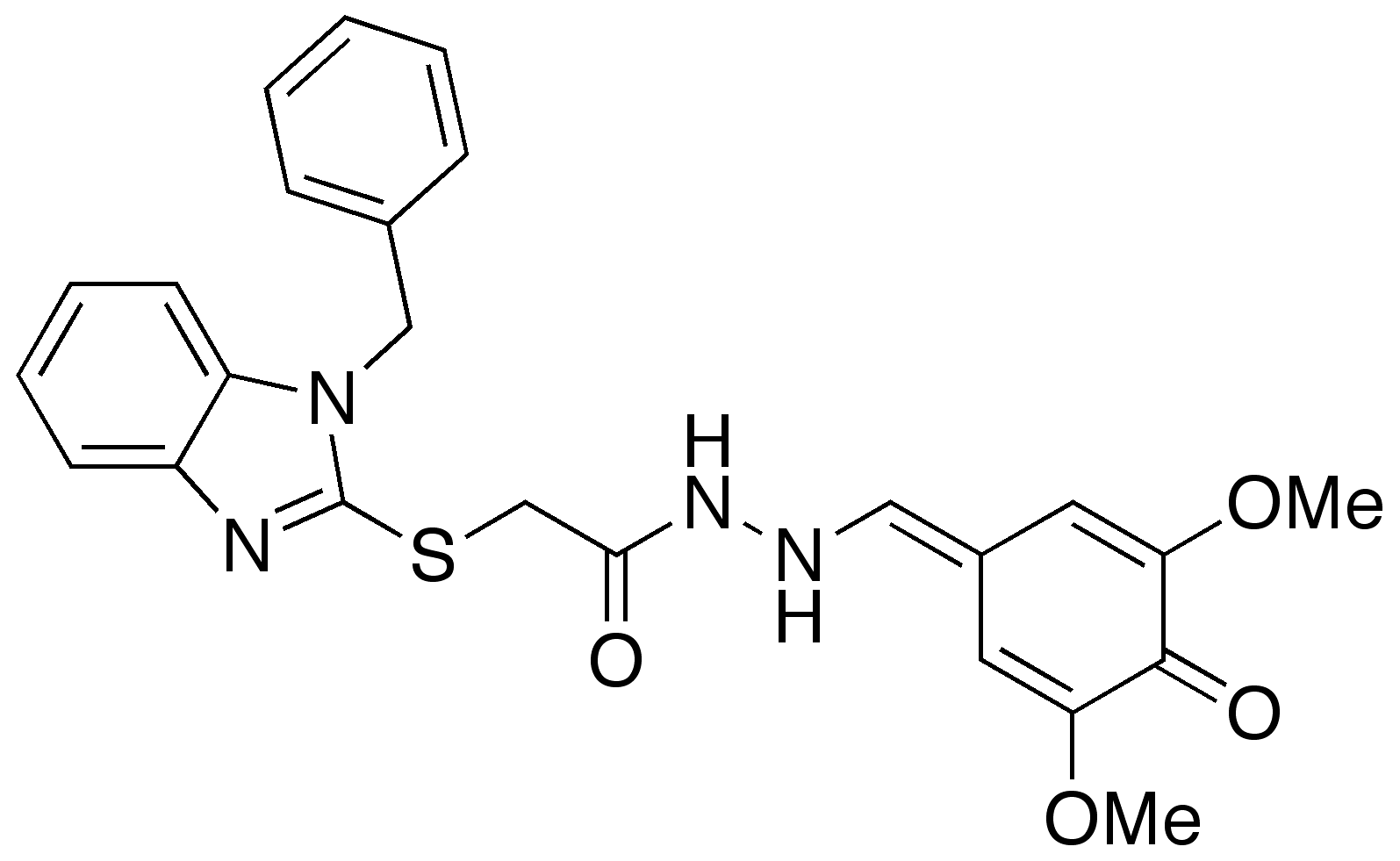 | Yes | --------- | BAS01809294 |
| NF2008 | 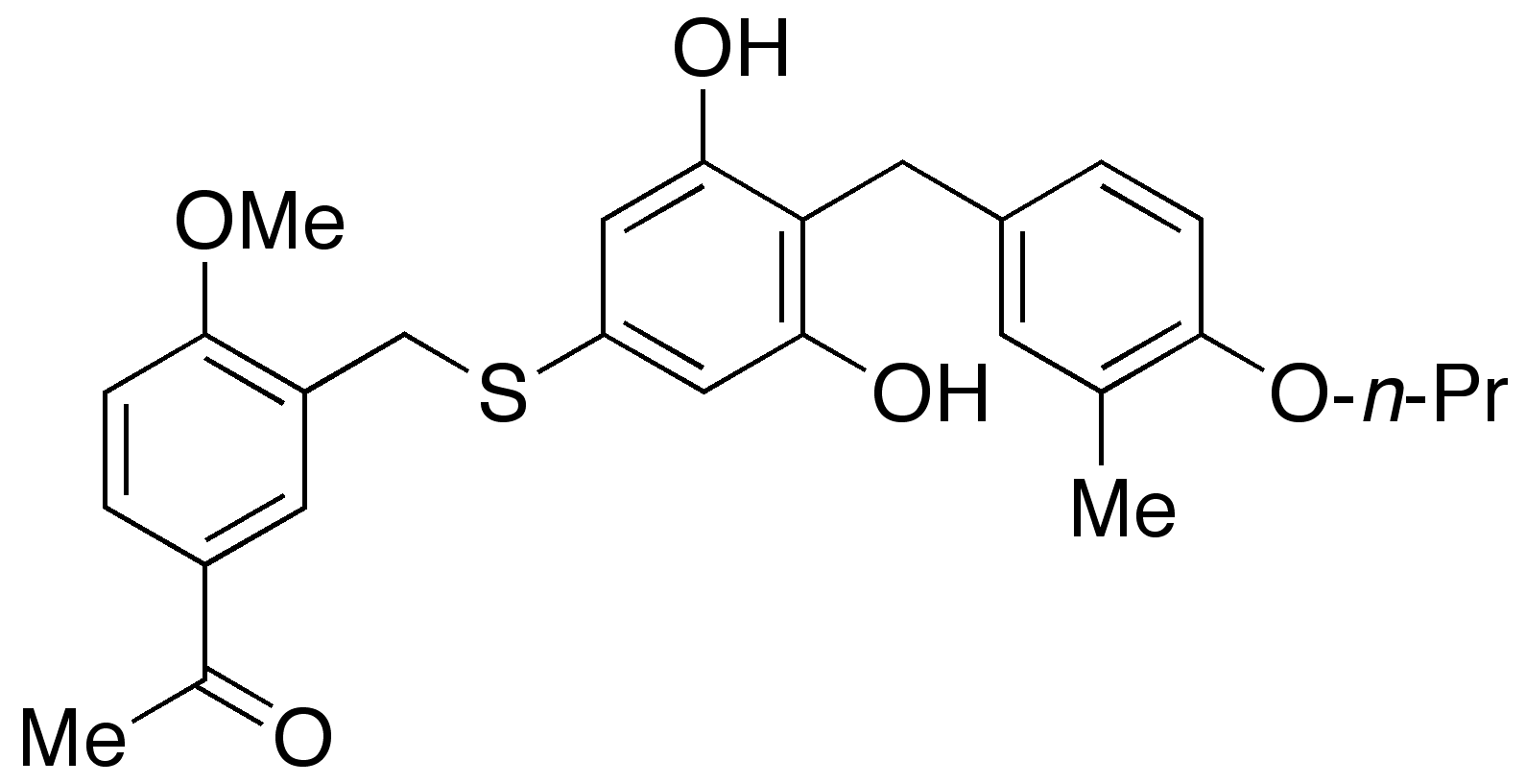 | Yes | --------- | BAS02141425 |
| NF2009 | 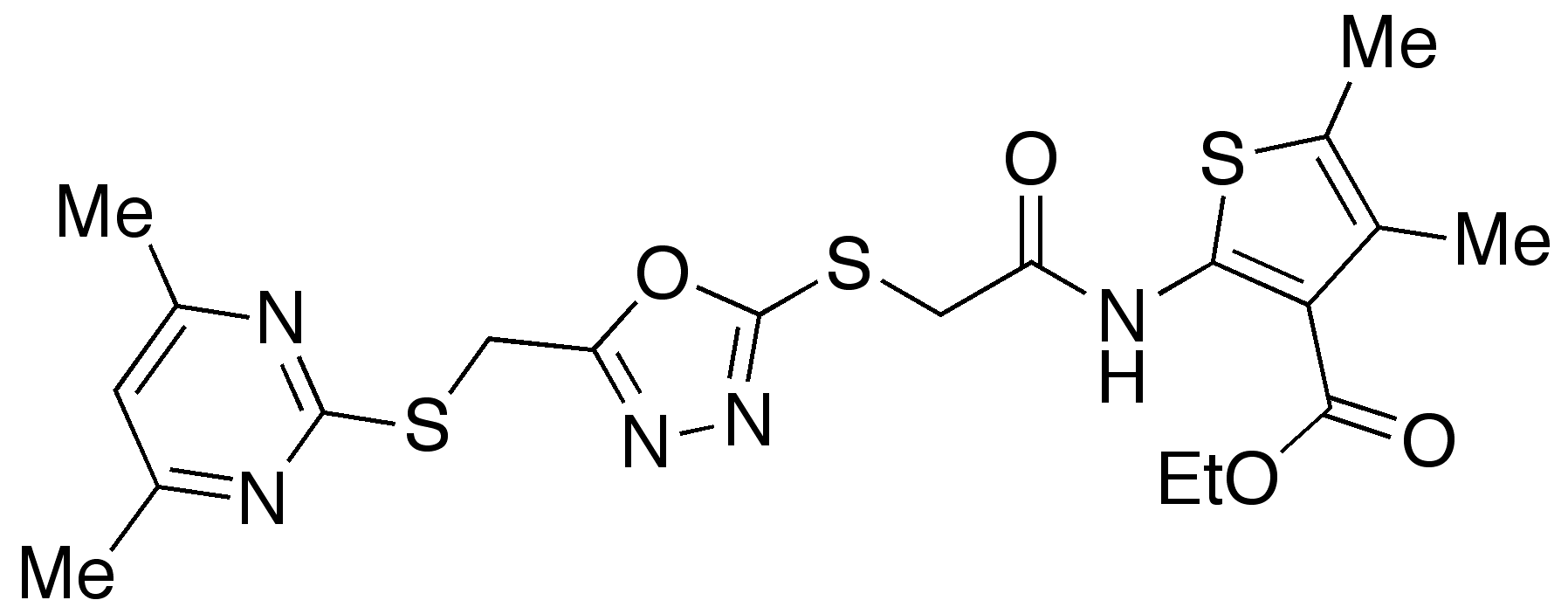 | Yes | --------- | BAS02323774 |
| NF2010 | 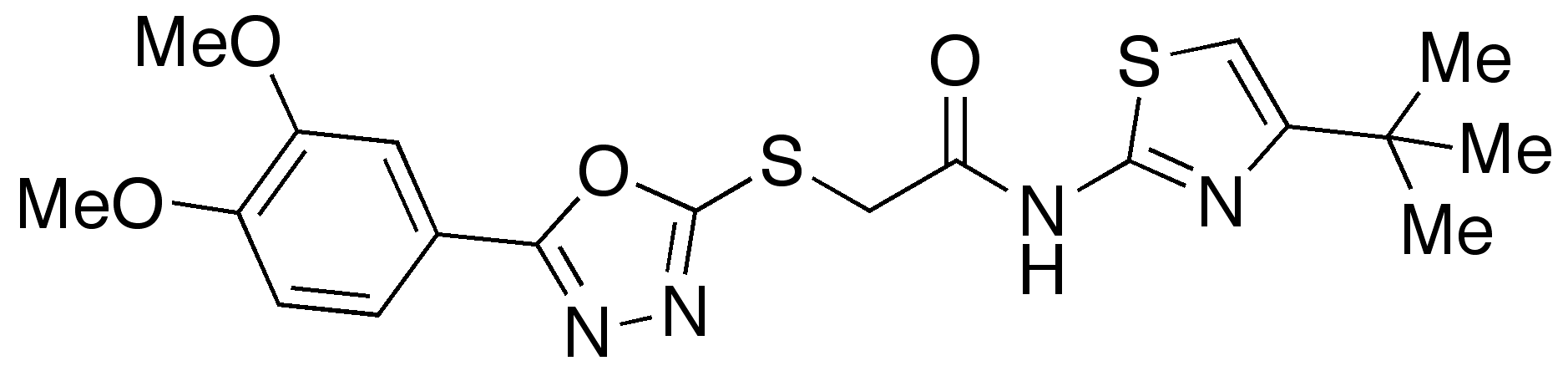 | No | 24 ± 6% | BAS10851825 |
| NF2011 | 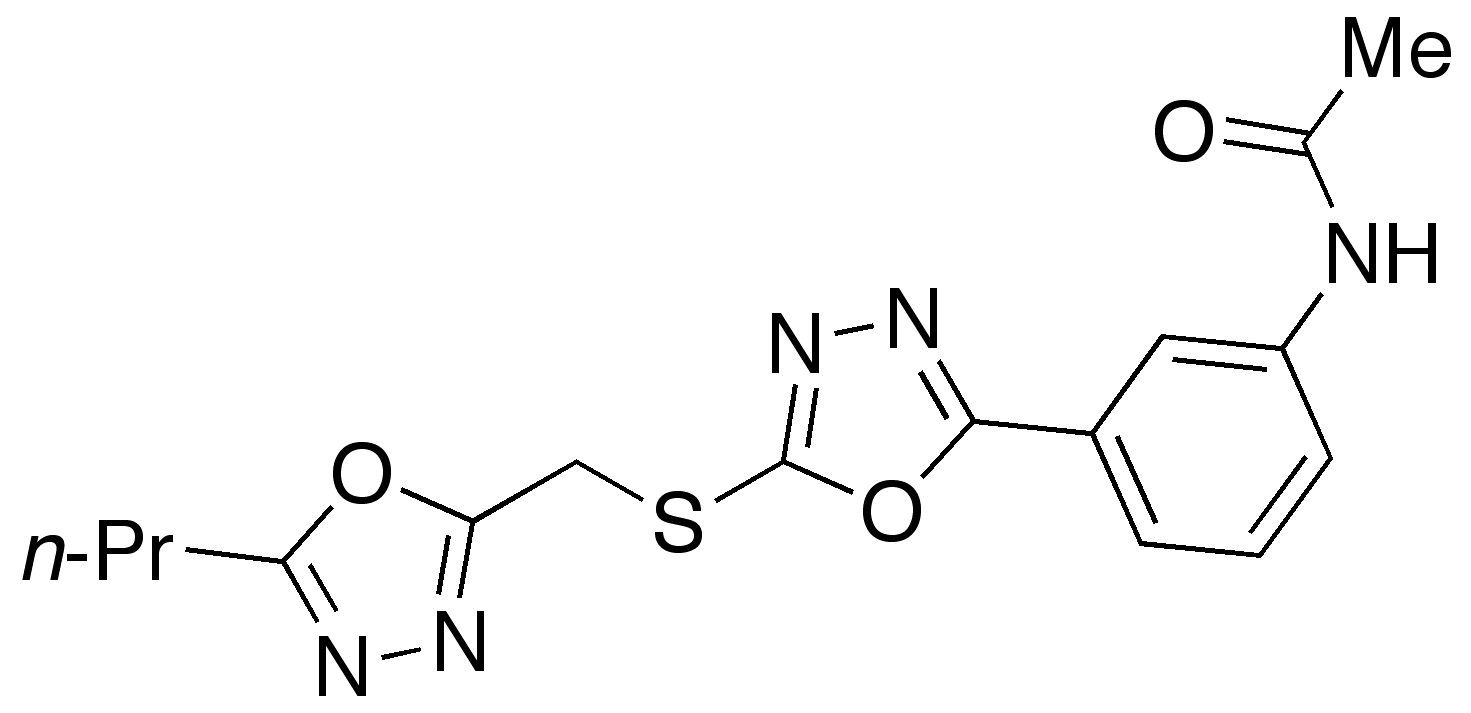 | No | 17 ± 7% | BAS13166183 |
| NF2012 | 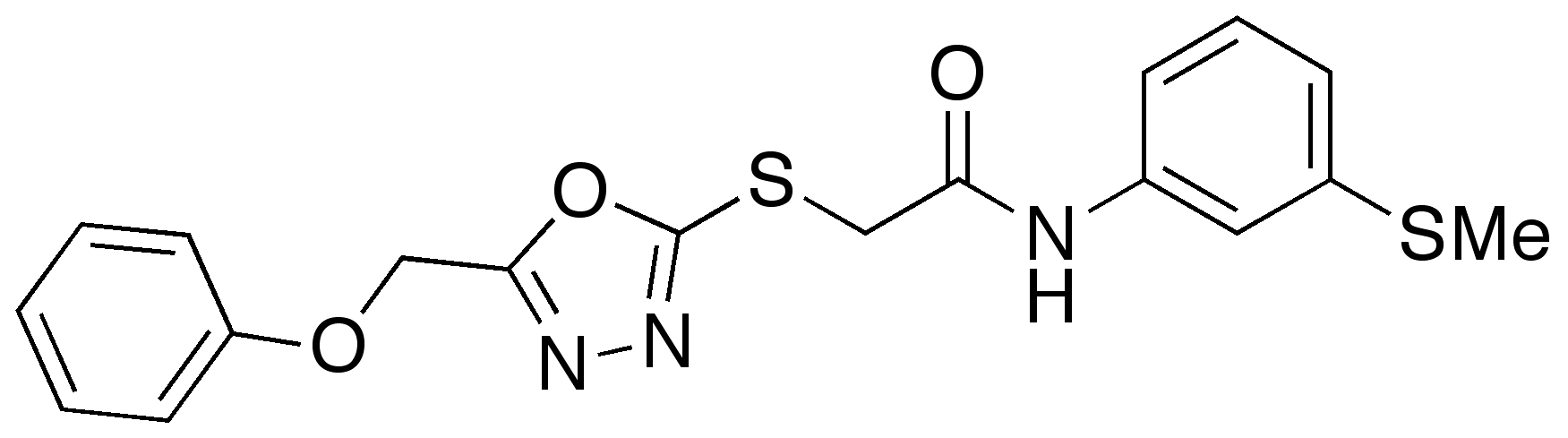 | Yes | --------- | BAS13407452 |
| NF2013 | 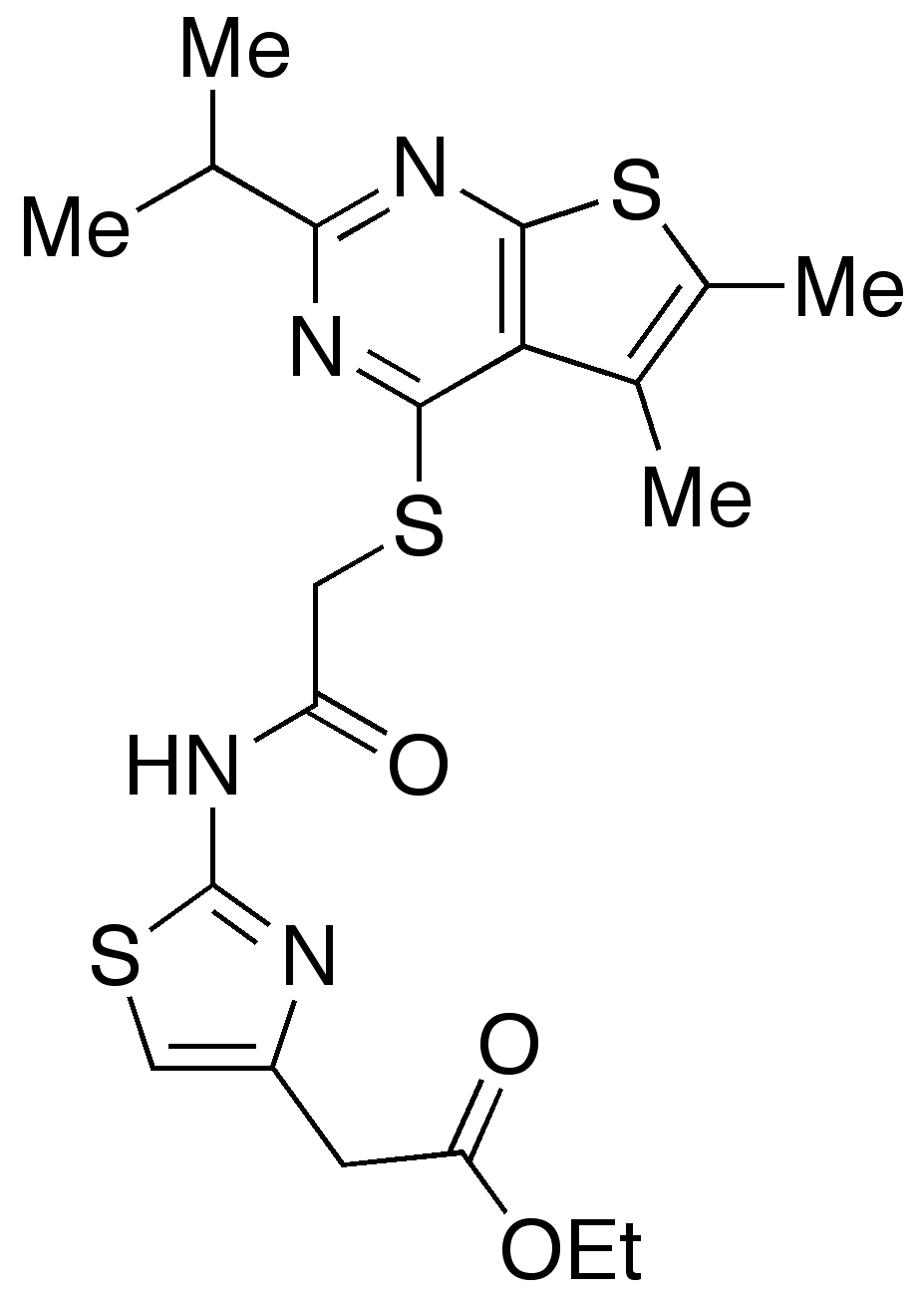 | Yes | --------- | ASN04890505 |
| NF2014 | 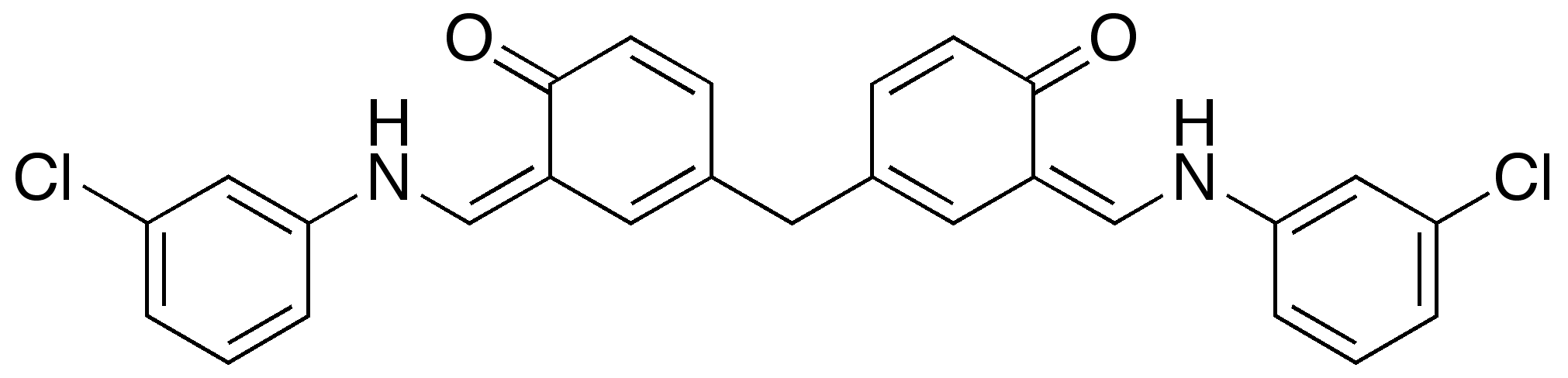 | Yes | --------- | BAS00395467 |
| NF2015 | 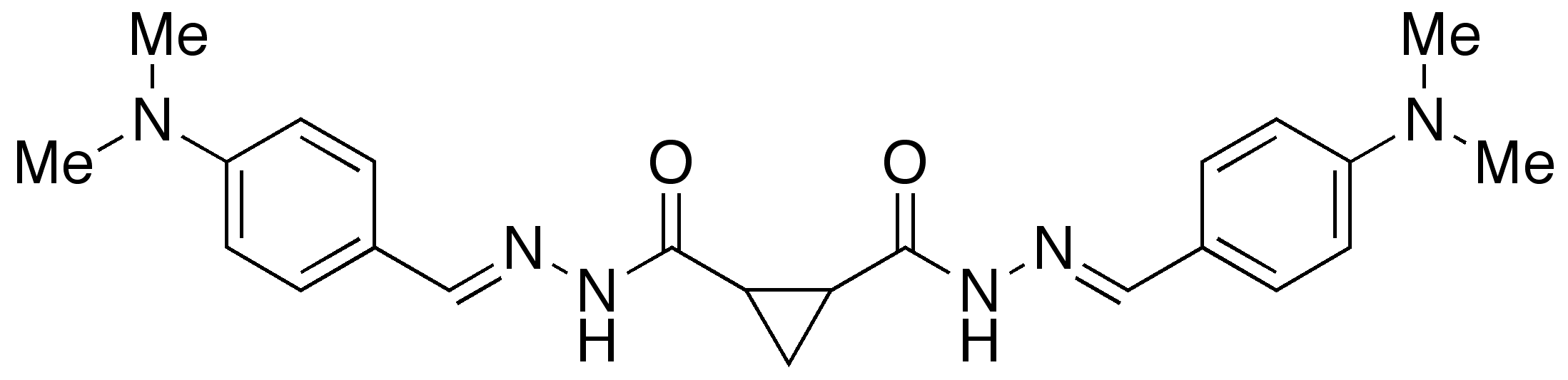 | No | 32 ± 5% | BAS00023895 |

*^a^*Horseradish peroxidase 2 µM was exposed to H_2_O_2_ 0.25 µM in sodium phosphate buffer pH 7.4 and 25 C°, in the presence of 2.5 µM LmTXNPx and 100 µM inhibitors. The residual activity of HRP has been calculated as ΔA/ΔA_0_ where ΔA_0_ is the difference in absorbance between HRP and compound I (ΔA_0_ =0.04) and ΔA is the difference in absorbance between HRP and compound I in the presence of LmTXNPx and inhibitors.

**Table S2.** Free-binding energies estimation

| Cpds | Docking Score^a^ | ΔG_bind_^b^ |
| --- | --- | --- |
| 1 | 70.49 | -109.87 |
| 2 | 71.94 | -121.40 |
| 3 | 71.22 | -105.32 |
| 4 | 77.78 | -117.43 |
| 5 | 72.92 | -120.91 |
| 6 | 81.02 | -128.82 |
| 7 | 78.46 | -121.69 |
| 8 | 73.41 | -107.43 |
| 9 | 76.74 | -112.26 |
| 10 | 73.56 | -119.58 |
| 11 | 72.21 | -110.18 |
| 12 | 78.91 | -128.96 |

^a^Docking score is referred to GoldScore scoring function available in GOLD software. ^b^ΔG_bind_ was calculated for all the compounds presented in this study by means of Prime MM/GBSA (Prime, Version 3.0, Schrödinger LLC, New York, NY 2011).

**Table S3.** Molecular properties for representative compounds selected by this study. The properties were calculated by means of QikProp (QikProp; version 3.4; Schrödinger, LLC: New York, 2011).

| **Compound** | **QPlogP^a^** | **QPlogS^b^** | **QPlogHERG^c^** | **QPPCaco^d^** | **QPlogBB^e^** | **QPPMDCK^f^** | **Human oral absorption^g^** |
| --- | --- | --- | --- | --- | --- | --- | --- |
| **1** | 4.556 | -5.523 | -7.951 | 140.209 | -0.990 | 118.364 | 2 |
| **3** | 3.539 | -5.183 | -7.647 | 29.142 | -1.857 | 27.528 | 2 |
| **5** | 5.213 | -6.508 | -7.894 | 140.209 | -0.827 | 340.183 | 1 |
| **6** | 5.937 | -7.112 | -8.664 | 103.344 | -1.295 | 80.704 | 1 |
| **7** | 3.801 | -4.162 | -7.365 | 112.319 | -0.999 | 51.509 | 3 |
| **8** | 3.896 | -4.489 | -7.865 | 103.357 | -1.010 | 47.081 | 3 |
| **12** | 4.949 | -5.889 | -7.119 | 128.380 | -1.115 | 59.514 | 3 |

^a^QPlogP predicted octanol/water partition coefficient (range or recommended value for 95% of known drugs -2-6.5); ^b^QPlogS predicted aqueous solubility in mol/dm3(range or recommended value for 95%of known drugs -6.5-0.5); ^c^QPlogHERG predicted IC_50_ value for blockage of HERG K^+^ channels (range or recommended below -5); ^d^QPPCaco predicted apparent Caco-2 cell permeability in nm/sec (range or recommended value for 95%of known drugs <25 poor >500 great); ^e^QPlogBB predicted brain/blood partition coefficient (range or recommended value for 95%of known drugs -3-1.2); ^f^QPPMDCK predicted apparent MDCK cell permeability in nm/sec (range or recommended value for 95% of known drugs <25 poor >500 great); ^g^Predicted qualitative human oral absorption: 1, 2, or 3 for low, medium and high, respectively. Range or recommended values are reported in QikProp user manual.

**Experimental procedures**

**2-Chloro-6,7-dimethoxyquinoline-3-carbaldehyde (21a). 20a** (1.0 g, 6.5 mmol) was slowly added to a mixture of acetic anhydride (3 mL) and a catalytic amount of H_2_SO_4_. The reaction was stirred under Ar atmosphere at 25 °C for 5 min then water was added. The aqueous phase was extracted with DCM (3 x 40 mL). The combined organic layers were washed with brine, dried over Na_2_SO_4_, filtered, and evaporated under reduced pressure to give the corresponding acetanilide as a brown solid (1.1 g, 86%). ^1^H NMR (300 MHz, CDCl_3_) δ 7.31 (d, *J* = 2.3 Hz, 1H), 6.94 – 6.68 (m, 2H), 3.86 (s, 3H), 3.85 (s, 3H), 2.15 (s, 3H). POCl_3_ (4.7 mL, 50.8 mmol) was added to dry DMF (1.4 mL, 17.5 mmol), at 0 °C under Ar atmosphere and stirred for 5 min. The acetanilide obtained in the previous step (1.0 g, 5.3 mmol) was added and reaction was stirred at 85 °C for 12 h. The reaction was poured on ice, filtered, and the solid was collected, washed with water, and dried. Compound **21a** (1.1 g, 88%) obtained as a yellow solid was used in the next step without any further purification. ^1^H NMR (300 MHz, CDCl_3_) δ 10.54 (s, 1H), 8.60 (s, 1H), 7.40 (s, 1H), 7.17 (s, 1H), 4.07 (s, 3H), 4.04 (s, 3H).

**2-Chloro-6-methoxyquinoline-3-carbaldehyde (21b).** Starting from **20b** (1.0 g, 8.1 mmol) the title compound was prepared following the procedure reported for compound **21a**. Compound **21b** (650 mg, 36%) obtained as a pale yellow solid, was used in the next step without any further purification. ^1^H NMR (300 MHz, CDCl_3_) δ 10.54 (s, 1H), 8.64 (s, 1H), 7.96 (d, *J* = 9.2 Hz, 1H), 7.51 (dd, *J* = 9.2, 2.8 Hz, 1H), 7.19 (d, *J* = 2.8 Hz, 1H), 3.95 (s, 3H).

**2-Chloroquinoline-3-carbaldehyde (21c).** Starting from **20c** (1.0 g, 10.8 mmol) the title compound was prepared following the procedure reported for compound **21a**. Compound **21c** (1.8 g, 88%) obtained as a pale brown solid, was used in the next step without any further purification. ^1^H NMR (300 MHz, CDCl_3_) δ 10.56 (s, 1H), 8.76 (s, 1H), 8.08 (d, *J* = 8.6 Hz, 1H), 7.99 (d, *J* = 8.2 Hz, 1H), 7.89 (t, *J* = 7.7 Hz, 1H), 7.66 (t, *J* = 7.5 Hz, 1H).

**6,7-Dimethoxy-2-oxo-1,2-dihydroquinoline-3-carbaldehyde (18a).** A solution of **21a** (1.1 g, 4.4 mmol) in acetic acid 70% (110 mL) was refluxed under Ar atmosphere for 12 h. Acetic acid was evaporated, a saturated solution of NaHCO_3_ was added till pH 7 and the mixture was extracted with DCM (3 x 40 mL). The combined organic layers were dried over Na_2_SO_4_, filtered, and evaporated under reduced pressure giving **18a** (765 mg, 75%) as a yellow solid, used in the next reaction without any further purification. ^1^H NMR (300 MHz, DMSO-*d*_6_) δ 12.07 (br s, 1H), 10.19 (s, 1H), 8.38 (s, 1H), 7.43 (s, 1H), 6.87 (s, 1H), 3.86 (s, 3H), 3.78 (s, 3H).

**6-Methoxy-2-oxo-1,2-dihydroquinoline-3-carbaldehyde (18b).** Starting from **21b** (200 mg, 0.9 mmol) the title compound was prepared following the procedure reported for compound **18a**. Compound **18b** (145 mg, 80%) obtained as pale yellow solid, was used in the next step without any further purification. ^1^H NMR (300 MHz, DMSO-*d*_6_) δ 12.12 (br s, 1H), 10.22 (s, 1H), 8.42 (s, 1H), 7.45 (d, *J* = 2.2 Hz, 1H), 7.37 – 7.16 (m, *J* = 2.5 Hz, 2H), 3.77 (s, 3H).

**2-Oxo-1,2-dihydroquinoline-3-carbaldehyde (18c).** Starting from **21c** (500 mg, 2.6 mmol) the title compound was prepared following the procedure reported for compound **18a**. Compound **18c** (382 mg, 85%) obtained as a pale brown solid, was used in the next step without any further purification. ^1^H NMR (400 MHz, DMSO-*d*_6_) δ 12.18 (br s, 1H), 10.20 (s, 1H), 8.47 (s, 1H), 7.88 (d, *J* = 7.8 Hz, 1H), 7.62 (t, *J* = 7.7 Hz, 1H), 7.32 (d, *J* = 8.3 Hz, 1H), 7.21 (t, *J* = 7.5 Hz, 1H).

**References**

1 Meth-Cohn, O., Narine, B. & Tarnowski, B. A versatile new sytnhesis of quinolines and related fused pyridines. Part II. *Tetrahedron Lett.* **33**, (1979).

2 Campiani, G. *et al.* Synthesis and pharmacological evaluation of potent and highly selective D3 receptor ligands: inhibition of cocaine-seeking behavior and the role of dopamine D3/D2 receptors. *J. Med. Chem.* **46**, 3822-3839, (2003).

3 Franceschini, S. Arylpiperazines as potential atypical antipsychotics - from design to pharmacokinetic optimization. *PhD Thesis in Pharmaceutical Sciences*, University of Siena, XXI Ciclo, (2009).

4 Gemma, S. *et al.* Optimization of 4-aminoquinoline/clotrimazole-based hybrid antimalarials: further structure-activity relationships, in vivo studies, and preliminary toxicity profiling. *J. Med. Chem.* **55**, 6948-6967, (2012).

5 Maramai, S. Design, synthesis and biological evaluation of novel and selective inhibitors of enzymes of the endocannabinoid system. *PhD Thesis in Chemical and Pharmaceutical Sciences*, University of Siena, XXV Ciclo, (2012).
